# CausalX-Net: a causality-guided explainable segmentation network for brain tumors

**DOI:** 10.3389/fmed.2025.1693603

**Published:** 2025-10-24

**Authors:** P. Suman Prakash, Patike Kiran Rao, M. Jahir Pasha, Ali Algarni, Manel Ayadi, Yongwon Cho, Yunyoung Nam

**Affiliations:** ^1^Department of Computer Science and Engineering-Artificial Intelligence, G Pullaiah College of Engineering and Technology, Kurnool, Andra Pradesh, India; ^2^Department of CSE (Data Science), Rajeev Gandhi Memorial College of Engineering and Technology, Nandyal, Andra Pradesh, India; ^3^Department of Computer Science & Engineering (AI&ML), G.Pulla Reddy Engineering College, Kurnool, Andra Pradesh, India; ^4^Informatics and Computer Systems Department, College of Computer Science, King Khalid University, Abha, Saudi Arabia; ^5^Center for Artificial Intelligence, King Khalid University, Abha, Saudi Arabia; ^6^Department of Information Systems, College of Computer and Information Sciences, Princess Nourah bint Abdulrahman University, Riyadh, Saudi Arabia; ^7^Department of Computer Science and Engineering, Soonchunhyang University, Asan, Republic of Korea

**Keywords:** CausalX-Net, brain tumor segmentation, causal effect (CE) maps, counterfactual explanations, explainable artificial intelligence (XAI), deep learning

## Abstract

Brain tumors represent a significant health challenge in India, with approximately 28,000 new cases diagnosed annually. Conventional deep learning approaches for MRI-based segmentation often struggle with irregular tumor boundaries, heterogeneous intensity patterns, and complex spatial relationships, resulting in limited clinical interpretability despite high numerical accuracy. This study introduces CausalX-Net, a causality-guided explainable segmentation network for brain tumor analysis from multi-modal MRI. Unlike purely correlation-based models, CausalX-Net leverages structural causal modeling and interventional reasoning to identify and quantify the causal influence of imaging features and spatial regions on segmentation outcomes. Through counterfactual analysis, the framework can provide clinically relevant “what-if” explanations, such as predicting changes in tumor classification if specific modalities, regions, or features are altered. Evaluated on the BraTS 2021 dataset, CausalX-Net achieved a Dice Similarity Coefficient of 92.5%, outperforming state-of-the-art CNN-based baselines by 4.3% while maintaining competitive inference efficiency. Furthermore, causal attribution maps and intervention-based sensitivity analyses enhance trust and transparency, offering radiologists actionable insights for diagnosis and treatment planning. This research demonstrates that integrating causal inference into segmentation not only improves accuracy but also delivers interpretable, decision-supportive explanations, representing a significant step toward transparent and reliable AI-assisted neuroimaging in clinical settings.

## 1 Introduction

**Figure 1:**
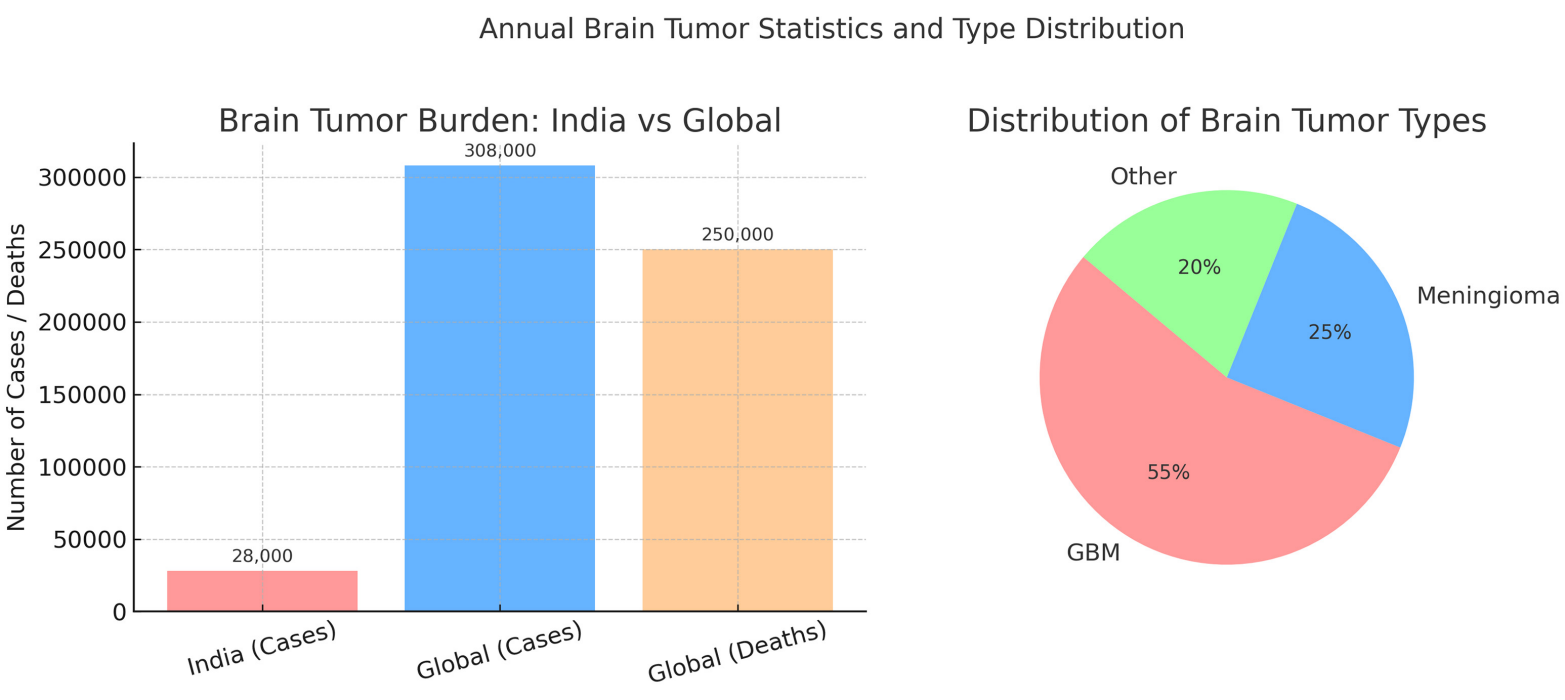
Annual brain tumor statistics for India and worldwide, including distribution of tumor types.

Brain tumors pose a significant global health burden, affecting individuals across all age groups. According to the Indian Council of Medical Research (ICMR), over 28,000 new brain tumor cases are diagnosed annually in India, with glioblastoma multiforme (GBM) being the most aggressive and lethal subtype (1). Globally, more than 308,000 new cases of central nervous system (CNS) tumors were reported in 2020, with over 250,000 deaths attributed to malignant brain tumors (2) (Figure 1). The 5-year survival rate for high-grade tumors such as GBM remains dismally low, often below 5%, primarily due to late detection and limited treatment options. Brain tumors are broadly classified into primary tumors (originating in the brain) and secondary tumors (metastases from other cancers). Primary tumors can be benign (non-cancerous) or malignant (cancerous). The World Health Organization (WHO) classifies brain tumors into low-grade (Grade I–II) and high-grade (Grade III–IV) tumors based on their growth rate and aggressiveness (3). The most common malignant brain tumor, GBM, has a median survival time of just 12–15 months despite aggressive treatment involving surgery, radiotherapy, and chemotherapy. In India, delayed diagnosis due to lack of awareness, limited access to MRI facilities, and financial constraints further critical patient outcomes. Early and accurate detection of brain tumors through automated MRI analysis could significantly improve prognosis by enabling timely intervention and personalized treatment strategies.

Magnetic Resonance Imaging (MRI) is the gold standard for diagnosing and characterizing brain tumors due to its excellent soft tissue contrast and multi-planar imaging capabilities. Different MRI sequences provide complementary information about tumor composition, which is essential for accurate segmentation and classification (4). T1-weighted (T1W) MRI offers detailed anatomical structure but has limited tumor contrast. T2-weighted (T2W) MRI highlights fluid-filled regions, including peritumoral edema, but lacks specificity. FLAIR (Fluid Attenuated Inversion Recovery) MRI suppresses cerebrospinal fluid (CSF) signals, making edema more distinguishable from normal brain tissue (5). T1-Contrast Enhanced (T1CE) MRI utilizes contrast agents to highlight the tumor's enhancing core, aiding in clear boundary delineation. Multi-modal fusion of these sequences is crucial for deep learning-based segmentation, as each modality provides unique tumor-related features. The BraTS dataset, a widely used benchmark for brain tumor segmentation, includes T1, T2, FLAIR, and T1CE MRI modalities to facilitate multi-modal learning (6). Despite the advantages of MRI, manual tumor segmentation is labor-intensive and prone to variability due to tumor heterogeneity in shape, size, and intensity, as well as low inter-observer agreement among radiologists. Automated segmentation using deep learning offers consistent, rapid, and accurate tumor delineation, making it a promising clinical tool. Deep learning, particularly Convolutional Neural Networks (CNNs), has achieved state-of-the-art results in medical image segmentation. The U-Net architecture (7), with its encoder–decoder structure and skip connections, remains one of the most widely used frameworks. Variants such as 3D U-Net, Attention U-Net, and Transformer-based architectures have improved contextual modeling and reduced false positives (8). Nevertheless, these models are primarily optimized for prediction accuracy and often rely on correlation-based feature attribution, which offers limited insight into the underlying decision-making process. This lack of interpretability reduces clinician trust in AI-assisted diagnostics, especially in critical applications like neuro-oncology.

Recent studies have explored Explainable AI (XAI) methods, including model-agnostic approaches (e.g., SHAP, LIME), gradient-based attribution (e.g., Grad-CAM), and attention-based mechanisms, to enhance interpretability (4). While these methods can identify salient regions influencing a model's decision, they generally do not address causality—i.e., they can explain *what* features are correlated with the output, but not *why* a decision was made or *what* changes would alter the outcome. Causal inference offers a principled framework for uncovering cause–effect relationships through techniques such as structural causal models (SCM), do-calculus, and counterfactual reasoning (9). In medical imaging, this enables clinically relevant reasoning, such as: “*If the edema region intensity were reduced, would the lesion still be classified as malignant?”*

**Figure 2:**
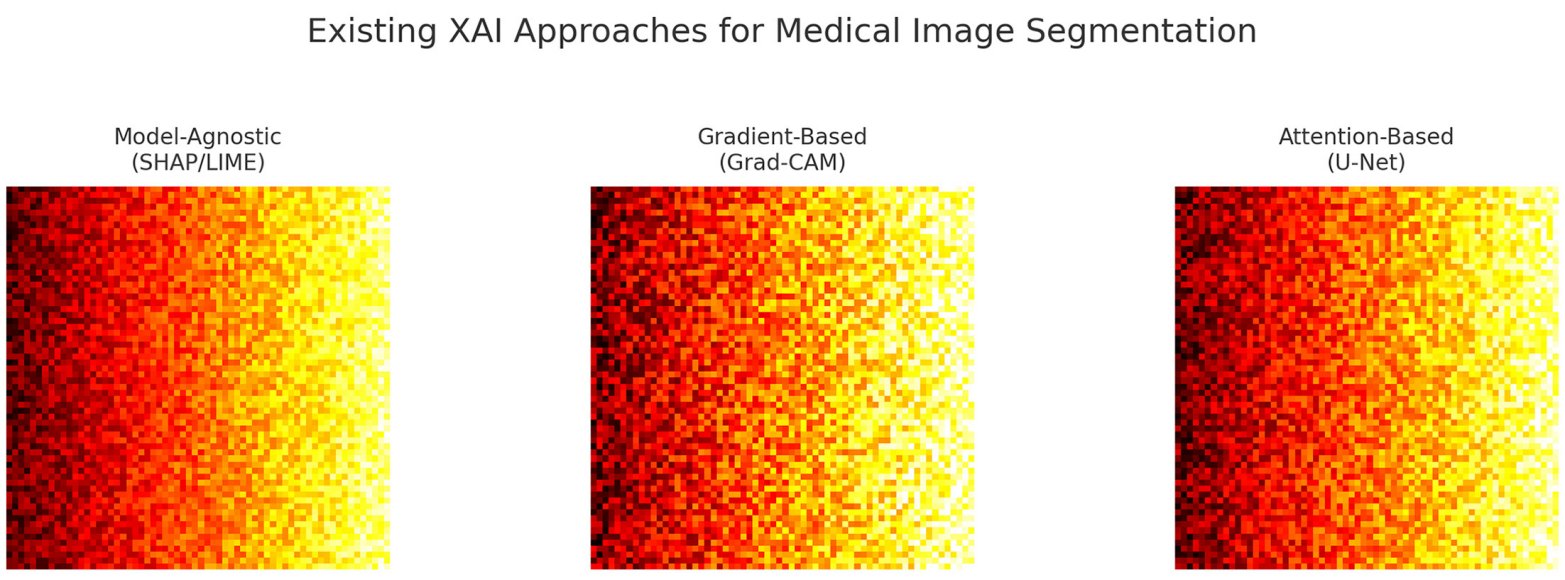
Examples of attribution maps from existing XAI methods, illustrating their focus on broad correlated regions rather than causally relevant tumor features.

In this study, we propose **CausalX-Net**, a causality-guided explainable segmentation network for brain tumor analysis from multi-modal MRI. CausalX-Net integrates a structural causal modeling layer into a high-performance 3D segmentation backbone, enabling both precise tumor delineation and interpretable, intervention-based reasoning. The causal layer models dependencies between imaging modalities, extracted features, and segmentation outputs, allowing counterfactual analyses that quantify how modifications in specific regions or features would alter predictions. Figure 2 illustrates the limitations of existing XAI methods, which often highlight broad correlated areas instead of causally relevant tumor regions. By shifting from correlation-driven to causation-aware segmentation, CausalX-Net bridges the gap between high-performance AI and trustworthy, clinically meaningful decision support.

### 1.1 Contributions and organization

Our work advances causal reasoning in brain tumor segmentation beyond prior methods (10, 11) through the following key contributions:

- We introduce **CausalX-Net**, the first causal segmentation framework embedding a structural causal model (SCM) into a 3D U-Net with explicit neuroimaging priors (e.g., enforcing known modality–region links such as FLAIR → ED, T1CE → ET).
- We design a novel **interventional training strategy with modality dropout**, enabling robust causal disentanglement under missing-modality conditions.
- We develop a **counterfactual auditing pipeline** for clinical error analysis, which quantifies voxel-wise causal effects and localizes spurious correlations - a feature absent in prior causal segmentation works.
- We demonstrate that these causal interventions deliver not just superior Dice/HD95 performance, but **practical clinical benefits**: enhanced boundary confidence for tumor margin planning and robust predictions under acquisition noise or incomplete scans.

The remainder of this paper is organized as follows: Section 2 reviews literature on explainable AI, causal inference, and brain tumor segmentation. Section 3 details the CausalX-Net architecture and causal reasoning mechanisms. Section 4 presents experimental results and interpretation case studies, followed by a conclusion of implications in Section 5.

## 2 Related work

### 2.1 Conventional brain tumor segmentation

Early brain tumor segmentation relied on classical image processing techniques such as intensity thresholding, region growing, morphological operations, and atlas registration (12, 13). These methods assumed consistent tissue intensity distributions and anatomical priors, which proved inadequate for the heterogeneity of glioblastomas. Thresholding failed under overlapping intensities and infiltrative margins; region growing required manual seeds and struggled with irregular boundaries; atlas-based methods degraded under mass effects, brain shifts, and large deformations. While computationally efficient and interpretable, these approaches lacked robustness to morphological heterogeneity, intensity inhomogeneity, and acquisition artifacts, and could not adapt to partial volume effects—necessitating a shift to data-driven learning-based methods.

### 2.2 CNN-based deep learning models

CNNs revolutionized medical segmentation by enabling hierarchical feature learning. U-Net (3) introduced the encoder–decoder paradigm with skip connections for multi-scale fusion and precise localization. V-Net (14) extended this to 3D, leveraging volumetric convolutions and Dice-based loss to address class imbalance, capturing inter-slice dependencies at higher computational cost. Myronenko (5) incorporated variational autoencoder (VAE) regularization for robust representation learning. DenseNet-based models (15) improved gradient flow and feature reuse through dense connectivity. Attention U-Net (16) integrated attention gates to focus on tumor-relevant regions. Despite state-of-the-art performance, CNNs suffered from limited receptive fields, hindering modeling of long-range dependencies and global anatomical context essential for accurate tumor boundary delineation.

### 2.3 Transformer-based models

Vision Transformers addressed CNN limitations by modeling long-range dependencies via self-attention. TransBTS (17) hybridized CNN encoders with Transformer blocks to combine local feature extraction and global context modeling. Swin-UNet (18) introduced shifted window attention to balance global modeling with computational efficiency. UNETR (19) replaced the encoder with a pure Transformer, processing images as patch sequences and achieving state-of-the-art BraTS performance. Transformers excel at capturing spatial relationships and multi-modal fusion but demand large datasets, heavy computation, and offer limited interpretability compared to CNNs.

### 2.4 Graph and hypergraph learning approaches

Graph-based methods modeled non-Euclidean brain structures as nodes and edges, capturing complex relationships beyond grid-based CNNs. Feng et al. (20) proposed multi-modal hypergraph learning to encode higher-order relationships among T1, T1ce, T2, and FLAIR via superpixels and hyperedges built on spatial, intensity, and cross-modal similarities. Zhao et al. (21) combined SLIC superpixels with graph convolutional networks (GCNs) for region-level message passing, preserving spatial topology. Extensions included dynamic graph construction, attention-weighted edges, and multi-scale graph hierarchies to capture local and global context. Despite strong performance in multi-modal modeling, graph-based methods face high computational cost, complex implementation, and poor GPU efficiency, slowing clinical adoption.

### 2.5 Explainable AI in medical imaging

Rising model complexity spurred explainable AI (XAI) to enhance trust and regulatory compliance. Gradient-based saliency maps (22) identified influential pixels but produced noisy, hard-to-interpret results; guided backprop and integrated gradients improved clarity but remained correlational. Grad-CAM (23) localized class-relevant regions using feature map gradients, aiding multi-class tumor segmentation yet still correlation-based. Model-agnostic methods like LIME (24) and SHAP (25) perturbed inputs to estimate local feature importance, but similarly captured associations rather than causal mechanisms—limiting clinical reliability where spurious correlations can mislead models.

### 2.6 Causal inference-based approaches

Causal inference emerged to improve robustness and interpretability by modeling cause–effect mechanisms rather than associations. Structural Causal Models (SCMs) (9) formalize variables and directed edges in causal graphs, enabling reasoning across Pearl's hierarchy of association, intervention, and counterfactuals. Karimi et al. (11) generated counterfactuals for diagnostic systems, identifying minimal patient changes altering predictions for actionable insights. Zhang et al. (10) integrated intervention-based causal reasoning into segmentation, linking modality features to anatomical structures to improve robustness and generalization. Recent work explores causal representation learning and mediation analysis to reveal predictive mechanisms. Despite promise, causal methods remain underexplored in brain tumor segmentation due to implementation complexity, scarce interventional data, and validation challenges. Unlike prior causal or counterfactual reasoning studies (10, 11), which either generated *post-hoc* counterfactuals or modeled interventions outside the segmentation loop, our approach integrates structural causal reasoning directly into the predictive pipeline. By embedding an SCM branch within the network and coupling it with interventional training, CausalX-Net enables voxel-level causal attribution during segmentation itself. This design allows simultaneous prediction and causal explanation, providing actionable insights for clinical planning rather than purely retrospective analysis.

### 2.7 Research gaps

Tables 1, 2 summarize key contributions, trends, and limitations across major brain tumor segmentation paradigms. The field has evolved from conventional to deep learning-based methods, achieving substantial gains in automation and accuracy at the expense of computational cost and interpretability. Classical techniques (thresholding, region growing, atlas-based) (12, 13) offered efficiency and transparency but failed on heterogeneous tumors, intensity inhomogeneity, and partial volume effects. CNN-based models (3, 5, 14–16) introduced automated feature learning, skip connections, dense connectivity, and attention, delivering strong performance but suffering from high computational demands, limited receptive fields, and reduced explainability. 3D extensions improved volumetric context modeling yet further increased resource requirements.

**Table 1:** Comparative analysis of brain tumor segmentation and interpretability methods (part 1).

**Study**	**Dataset**	**Method**	**Key contributions**	**Critical limitations**
Menze et al. (2015) (12)	BraTS	Atlas-based registration	Established benchmark; multi-modal integration; anatomical priors	Poor generalization; deformation sensitivity; manual intervention
Clark et al. (2013) (13)	TCIA GBM	Intensity thresholding	Computational efficiency; interpretable approach	Heterogeneity failure; intensity inhomogeneity sensitivity
Ronneberger et al. (2015) (3)	Medical datasets	U-Net architecture	Skip connections; precise boundary delineation; small dataset effectiveness	Limited global context; class imbalance challenges
Milletari et al. (2016) (14)	Medical MRI	V-Net (3D CNN)	Volumetric processing; Dice loss innovation; 3D context capture	Memory constraints; annotation requirements; computational overhead
Myronenko (2018) (5)	BraTS	U-Net + VAE regularization	Multi-task learning; improved robustness; regularized representation	Architectural complexity; hyperparameter sensitivity; training difficulty
Li et al. (2018) (15)	BraTS	DenseNet segmentation	Feature reuse; gradient flow optimization; parameter efficiency	GPU memory demands; training instability; implementation complexity
Oktay et al. (2018) (16)	Medical MRI	Attention U-Net	Spatial attention mechanisms; focused learning; adaptive weighting	Limited attention interpretability; computational overhead
Wang et al. (2022) (17)	BraTS	TransBTS hybrid	CNN-Transformer synergy; global dependency modeling; multi-scale integration	Computational complexity; large dataset requirements; training difficulty
Cao et al. (2021) (18)	BraTS	Swin-UNet	Hierarchical attention; shifted window efficiency; multi-scale modeling	Dataset size dependency; architectural complexity; limited validation

**Table 2:** Comparative analysis of brain tumor segmentation and interpretability methods (part 2).

**Study**	**Dataset**	**Method**	**Key contributions**	**Critical limitations**
Hatamizadeh et al. (2022) (19)	BraTS	UNETR (pure transformer)	Full Transformer encoder; SOTA performance; patch-based processing	Resource intensity; interpretability loss; scaling challenges
Feng et al. (2019) (20)	BraTS	Hypergraph learning	Higher-order multi-modal relationships; complex dependency modeling	Scalability constraints; implementation complexity; GPU incompatibility
Zhao et al. (2022) (21)	BraTS	SLIC + GCN	Region-level processing; graph-based reasoning; noise reduction	Superpixel quality dependency; irregular processing; scalability issues
Zhang et al. (2025) (22)	ImageNet	Saliency maps	Gradient-based visualization; model-agnostic application	Noise susceptibility; spatial incoherence; clinical inapplicability
Selvaraju et al. (2017) (23)	Medical datasets	Grad-CAM	Class-specific visual explanations; localization capability	Correlation-only insights; resolution limitations; causal blindness
Albalawi et al. (2016) (8)	Multiple	LIME	Model-agnostic explanations; local fidelity; intuitive interpretation	Local scope limitation; result instability; global insight absence
Xu et al. (2017) (26)	Multiple	SHAP framework	Unified feature attribution; theoretical foundation; mathematical properties	Computational expense; approximation errors; causal ignorance
Karimi et al. (2021) (11)	Medical tabular	SCM + counterfactuals	Causal reasoning; actionable insights; what-if analysis	Structured data limitation; assumption dependency; validation gaps
Zhang et al. (2022) (10)	Medical MRI	Causal segmentation	Intervention-based reasoning; robustness improvement; mechanistic understanding	Early-stage development; limited validation; implementation barriers

Transformers (17–19) addressed CNNs' global context limitations via self-attention, with hybrid CNN–Transformer architectures showing practical superiority over pure forms. However, they require large datasets, extensive computation, and sacrifice interpretability. Graph-based methods (20, 21) capture complex non-Euclidean relationships and multi-modal dependencies but face scalability, GPU inefficiency, and implementation complexity, restricting clinical adoption.

Interpretability methods remain the widest gap: gradient-based saliency (22), Grad-CAM (23), and model-agnostic methods [LIME (24), SHAP (26)] provide only correlation-level insights, insufficient for clinical decision-making. Causal inference approaches (9–11) promise mechanistic understanding and robustness but remain early-stage, with limited validation and scarce interventional data.

Further gaps include: (i) lack of standardized evaluation protocols hindering fair comparisons, (ii) limited cross-dataset generalization analyses, (iii) minimal integration of uncertainty quantification into segmentation pipelines, and (iv) scarce real-world clinical validation of interpretability techniques. Future research should prioritize: (1) designing efficient yet accurate architectures for real-time clinical deployment, (2) advancing causal interpretability with rigorous clinical validation, (3) establishing standardized benchmarks and evaluation frameworks, (4) embedding uncertainty quantification for risk-aware predictions, and (5) performing large-scale multi-center studies to assess generalization across diverse cohorts.

## 3 Methodology

This section presents CausalX-Net, a novel causality-guided framework that addresses the fundamental limitations of correlation-based attribution methods in brain tumor segmentation. Our approach integrates a *Structural Causal Model* (SCM) within a robust 3D segmentation backbone, enabling principled interventional and counterfactual reasoning for mechanistic interpretability. Unlike conventional explainable AI methods that rely on statistical associations, CausalX-Net provides causal explanations by modeling the underlying data-generating process and enabling “what-if” scenario analysis crucial for clinical decision-making.

CausalX-Net departs from prior causal segmentation works (10, 11) through three key components: (i) a structural causal model (SCM) branch embedded with neuroimaging priors [Fluid-Attenuated Inversion Recovery (FLAIR) → edema (ED), T1-Contrast-Enhanced (T1CE) → enhancing tumor (ET)], (ii) an interventional training strategy with modality dropout to enforce robust causal disentanglement, and (iii) a counterfactual auditing pipeline for voxel-wise causal effect analysis and clinical error tracing.

### 3.1 Architecture

Given multi-modal MRI data *X* = {*X*^T1^, *X*^T2^, *X*^FLAIR^, *X*^T1CE^}, where each modality represents a 3D volume of dimensions *H*×*W*×*D*, our objective is to learn a mapping function that predicts voxel-wise segmentation labels *Y*∈{0, …, *C*−1}^*H*×*W*×*D*^. The class labels correspond to: background (0), necrotic/non-enhancing tumor core (NCR, 1), peritumoral edema (ED, 2), and enhancing tumor (ET, 3). The learning problem is formulated as:


$$\begin{array}{l} f_{\theta }:X\mapsto Y, \end{array}$$

where θ represents the complete set of trainable parameters encompassing both the segmentation backbone and the embedded causal reasoning components. The key innovation lies in decomposing this mapping into causally interpretable components that explicitly model the relationships between imaging modalities, latent feature representations, and segmentation outcomes. This decomposition enables principled interventional analysis and counterfactual reasoning, providing mechanistic explanations for model decisions.

**Figure 3:**
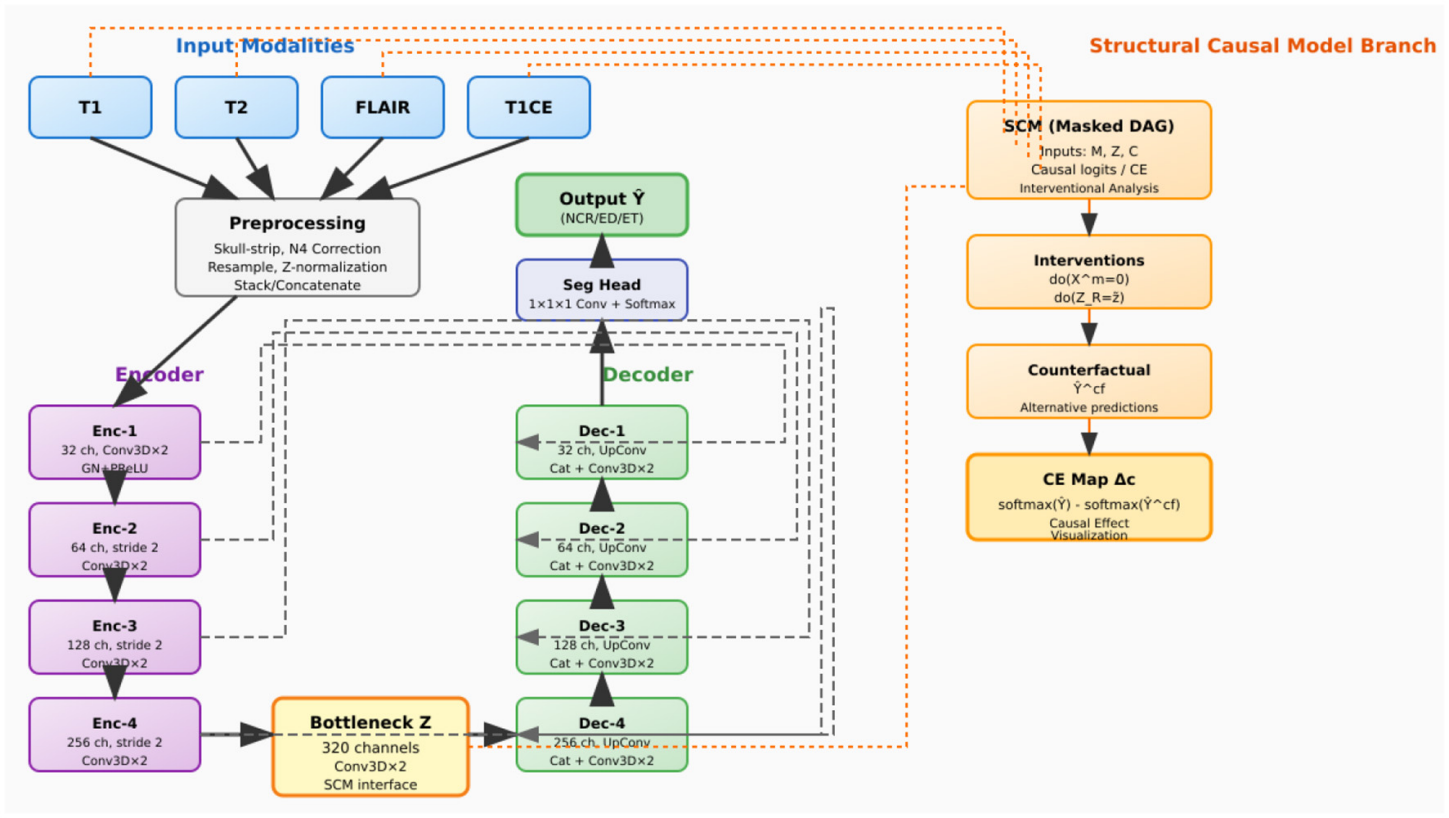
CausalX-Net (vertical layout, background-routed connectors). Encoder **(left)** builds latent Z; decoder **(right)** reconstructs labels with dashed, background routed skips to avoid overlaps. Far-right SCM branch receives modalities and Z, supports do-interventions, counterfactuals, and CE maps.

As illustrated in Figure 3, CausalX-Net employs a modular architecture comprising three interconnected components designed to balance segmentation performance with causal interpretability:

1. **3D segmentation backbone**: a state-of-the-art encoder-decoder architecture with residual connections and skip pathways for high-quality volumetric feature extraction and precise voxel-wise predictions (3, 5).
2. **Structural causal model (SCM) layer**: a learnable causal graph that explicitly models directed relationships among imaging modalities, latent feature representations, and segmentation labels, enabling principled causal reasoning.
3. **Interventional reasoning module**: a specialized component that executes do-calculus operations and counterfactual queries to generate causal attribution maps and mechanistic explanations.

This integrated design ensures that causal reasoning capabilities are embedded throughout the learning process rather than applied as *post-hoc* explanations, resulting in more reliable and mechanistically grounded interpretations.

### 3.2 Data preprocessing

Preprocessing follows a standardized pipeline designed to ensure consistency across imaging protocols and enhance model robustness. Each modality undergoes skull-stripping using HD-BET, N4 bias field correction to mitigate intensity inhomogeneity, resampling to a common isotropic voxel spacing of 1.0mm^3^, spatial normalization through padding or cropping to a fixed grid size, and z-score intensity normalization based on brain tissue statistics.

**Figure 4:**
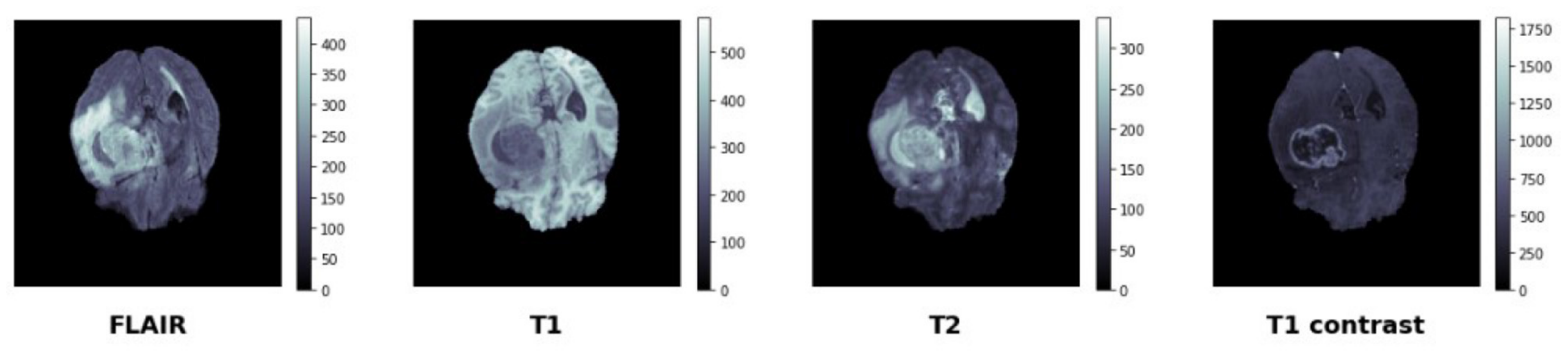
BraTS data set—manually segmented mask—target.

During training, we implement a comprehensive augmentation strategy that includes: (i) 3D spatial transformations (rotations up to ±15°, elastic deformations with displacement fields), (ii) intensity perturbations (Gaussian noise, gamma correction, intensity scaling), and (iii) *modality dropout*, where individual modalities are randomly ablated to probe causal robustness and prevent over-reliance on specific imaging sequences. This modality dropout strategy is particularly crucial for training the SCM to handle missing modalities and understand causal dependencies between different imaging contrasts as shown in Figure 4.

### 3.3 Segmentation

The segmentation backbone adopts a 3D U-Net-inspired encoder-decoder architecture with modern architectural improvements for enhanced feature learning and gradient flow. The detailed configuration is provided in Table 3, which specifies the layer-wise operations, channel dimensions, stride parameters, and skip connection arrangements.

**Table 3:** 3D segmentation architecture.

**Level**	**Operations**	**Channels**	**Stride**	**Skip connection**
Encoder-1	Conv3D × 2 (GN+PReLU)	32	1	To decoder-1
Encoder-2	Conv3D × 2 + Strided Conv	64	2	To decoder-2
Encoder-3	Conv3D × 2 + Strided Conv	128	2	To decoder-3
Encoder-4	Conv3D × 2 + Strided Conv	256	2	To decoder-4
Bottleneck	Conv3D × 2 + SCM Interface	320	1	To SCM layer
Decoder-4	UpConv + Concat + Conv3D × 2	256	–	From encoder-4
Decoder-3	UpConv + Concat + Conv3D × 2	128	–	From encoder-3
Decoder-2	UpConv + Concat + Conv3D × 2	64	–	From encoder-2
Decoder-1	UpConv + Concat + Conv3D × 2	32	–	From encoder-1
Output head	1 × 1 × 1 Conv + Softmax	*C* = 4	–	Final predictions

The architecture consists of:

- **Encoder blocks**: each level contains two consecutive Conv3D(3 × 3 × 3) → GroupNorm → PReLU operations with residual connections within blocks. Downsampling is achieved through strided convolutions with learnable parameters.
- **Decoder blocks**: transposed Conv3D layers perform learnable upsampling, followed by feature concatenation with corresponding encoder representations through skip connections. Two subsequent Conv3D layers with GroupNorm and PReLU activations refine the upsampled features.
- **Classification head**: a 1 × 1 × 1 Conv3D layer followed by softmax activation produces class probability distributions for each voxel.

The bottleneck latent representation *Z*∈ℝ^*H*^′ × *W*′ × *D*′ × *K* captures high-level semantic information and serves as the primary interface with the SCM layer. When interfacing with the SCM, *Z* is flattened to *Z*_♭_ while preserving spatial correspondence for voxel-wise causal reasoning.

### 3.4 Structural causal model (SCM) layer design

**Figure 5:**
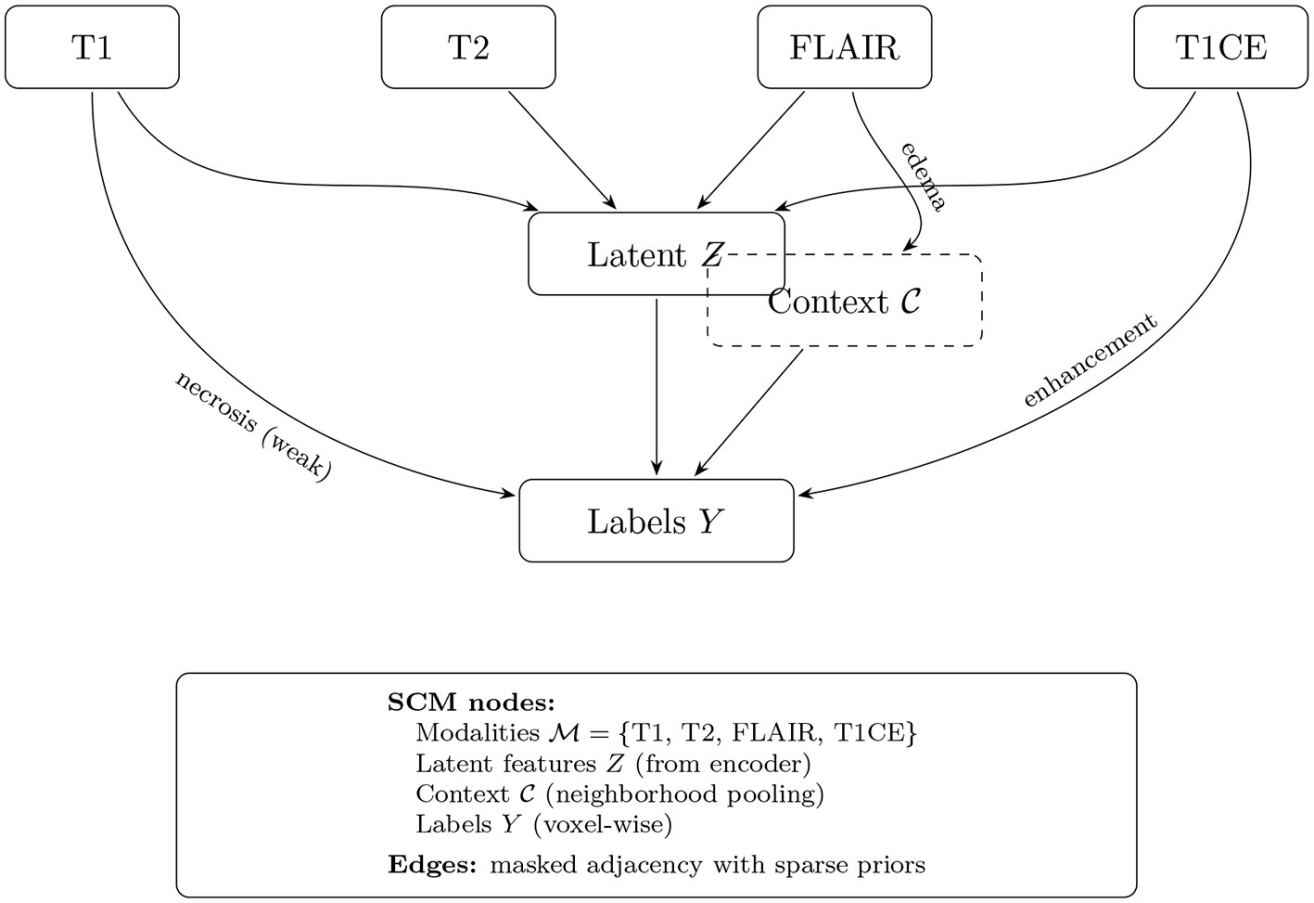
Structural causal model (SCM) in CausalX-Net. Directed edges encode domain-informed relations among MRI modalities, latent features, context, and labels. Masked adjacency and sparsity regularization enforce prior-consistent graphs while allowing data-driven refinement.

The SCM layer implements a learnable directed acyclic graph *G* = (*V, E*) where the vertex set *V* = {M, *Z, Y*} represents the causal variables: modality nodes M = {T1, T2, FLAIR, T1CE}, latent feature representations *Z*, and voxel-wise labels *Y*. The causal relationships are parameterized through structural equations:


$$\begin{array}{l} Z=f_{Z}\left(M,C_{Z}\right)+\epsilon_{Z},\;Y=f_{Y}\left(Z,M,C_{Y}\right)+\epsilon_{Y}, \end{array}$$

where C_*Z*_ and C_*Y*_ represent contextual information (e.g., 3D neighborhood features obtained through spatial pooling operations), and ϵ_*Z*_, ϵ_*Y*_ denote exogenous noise terms capturing unobserved confounders. The functions *f*_*Z*_ and *f*_*Y*_ are implemented as lightweight multi-layer perceptrons or 1 × 1 × 1 convolutional layers that operate on per-voxel features with shared weights across spatial locations. Figure 5 depicts the structural causal graph (DAG) used in CausalX-Net, highlighting directed dependencies among modalities, latent features, contextual cues, and voxel-wise labels.

We incorporate neuroimaging domain expertise through structured causal priors that reflect known relationships between imaging contrasts and tumor characteristics. Specifically, we enforce edges such as FLAIR → ED (FLAIR hyperintensity indicates edema), T1CE → ET (contrast enhancement reveals active tumor), and T1 → NCR (T1 hypointensity suggests necrosis). These priors are implemented through: (i) adjacency matrix masking that prevents biologically implausible causal relationships, and (ii) ℓ_1_ regularization penalties that encourage sparse, prior-consistent edge weights while allowing the model to learn data-driven refinements. Interventional reasoning forms the core of our causal explanation framework. An intervention do(*V* = *v*) replaces the structural equation of variable *V* with the constant value *v* and propagates the effects through the causal graph according to Pearl's do-calculus (27). We implement three complementary families of interventions:

1. **Modality-level interventions**: complete ablation do(*X*^*m*^ = **0**) or intensity scaling do(*X*^*m*^ = α*X*^*m*^) to assess modality importance and redundancy.
2. **Feature-level interventions**: regional feature clamping $\mathrm{do}\left(Z_{R}=\tilde{z}\right)$ for spatial regions L (e.g., tumor boundary zones) to understand local feature contributions.
3. **Lesion-specific interventions**: targeted intensity modifications within clinician-defined regions of interest to test model sensitivity and clinical relevance.

For a target tumor class *c*, the *causal effect* (CE) map quantifies the impact of each intervention:


$$\begin{array}{l} {\text{Δ}}_{c}\left(\text{x}\right)={\mathrm{softmax}}_{c}f_{Y}\left(Z,M\right)\left(\text{x}\right)-{\mathrm{softmax}}_{c}f_{Y}\left(Z,M\right)\mathrm{do}\left(\cdot \right)\left(\text{x}\right), \end{array}$$

where **x** denotes spatial coordinates. Positive values in Δ_*c*_ indicate regions where the intervention reduces class-*c* probability, providing voxel-wise causal attribution maps that can be overlaid on anatomical images for clinical interpretation. Counterfactual analysis addresses the clinically relevant question: “What would the segmentation outcome be if the imaging characteristics were different?” We implement the three-step Abduction-Action-Prediction (AAP) procedure (9):

1. **Abduction**: infer the exogenous noise terms ${\hat{\epsilon }}_{Z}$ and ${\hat{\epsilon }}_{Y}$ from the observed data (*X, Y*) by inverting the structural equations *f*_*Z*_ and *f*_*Y*_. This is achieved through either iterative optimization (single Newton step for computational efficiency) or amortized inference using trained encoder networks.
2. **Action**: apply a hypothetical intervention [e.g., do(*X*^T1CE^ = **0**) to simulate contrast agent absence] to the causal graph structure.
3. **Prediction**: generate counterfactual predictions Ŷ^cf^ under the modified graph using the inferred exogenous variables $\left({\hat{\epsilon }}_{Z},{\hat{\epsilon }}_{Y}\right)$, ensuring consistency with the original unobserved factors.

**Figure 6:**
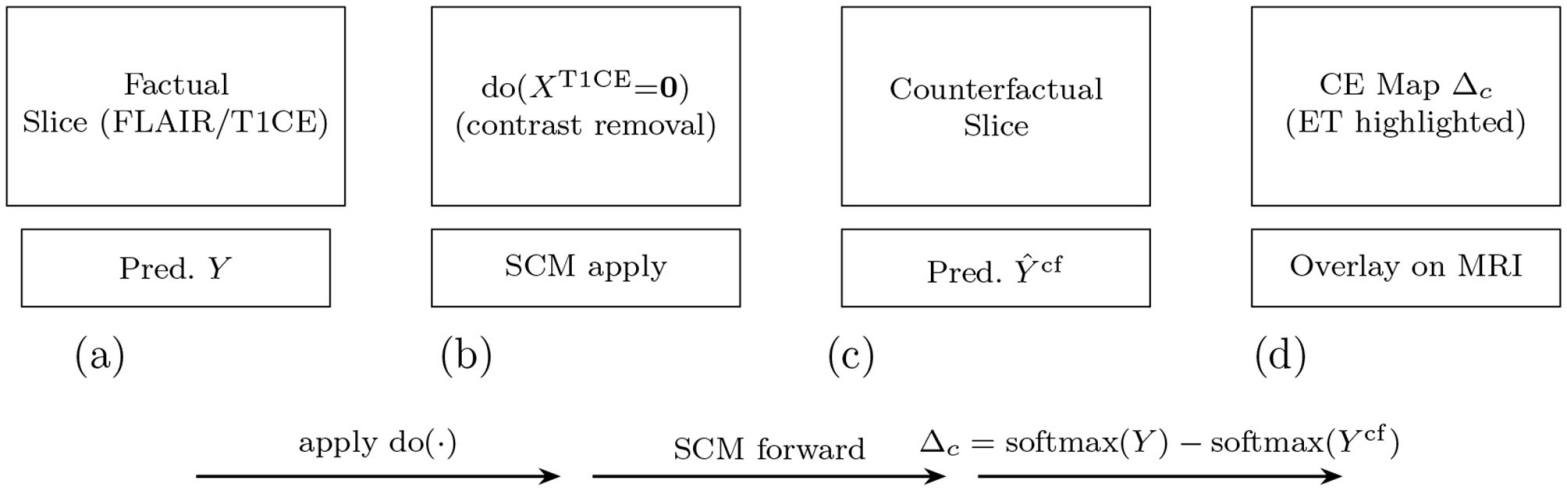
Counterfactual reasoning pipeline: **(a)** factual prediction (left), **(b)** intervention removing the T1CE modality via do(XT1CE = 0), **(c)** counterfactual prediction, and **(d)** resulting causal effect (CE) map. In this example, removing T1CE caused the enhancing tumor (ET) region to disappear, confirming its causal dependence on T1CE signal–an insight useful for treatment planning.

This procedure generates counterfactual segmentation masks and corresponding CE maps that provide clinicians with mechanistic explanations in the form of actionable “what-i” scenarios, facilitating treatment planning and diagnostic confidence assessment. An exemplar case is illustrated in Figure 6, showing the factual prediction, an intervention on T1CE via do(*X*^T1CE^ = **0**), the resulting counterfactual prediction, and the corresponding causal effect (CE) map.

### 3.5 Multi-objective training framework

The training objective combines segmentation accuracy with causal consistency through a carefully balanced composite loss function:


$$\begin{array}{l} L=L_{\text{Dice}}+\alpha L_{\text{CE}}+\beta L_{\text{Causal}}+\gamma L_{\text{Smooth}}. \end{array}$$

**Segmentation losses**: the dice coefficient loss M_Dice_ handles class imbalance while preserving spatial coherence, complemented by class-balanced multi-class cross-entropy L_CE_ for probability calibration.

**Causal consistency regularization**: the causal loss enforces adherence to domain priors and promotes sparse, interpretable causal graphs:


$$\begin{array}{l} L_{\text{Causal}}={\text{λ}}_{1}||\text{A}⊙\text{W}{||}_{1}+{\text{λ}}_{2}E_{\mathrm{do}}||{\text{Δ}}_{c}-{\text{Δ}}_{c}^{\text{prior}}{||}_{1}, \end{array}$$

where **W** represents learnable SCM edge weights, **A** is a binary mask encoding forbidden edges, and ${\text{Δ}}_{c}^{\text{prior}}$ encodes weak expectations about causal effects (e.g., FLAIR interventions should strongly affect edema predictions).

**Spatial regularization**: a boundary-preserving conditional random field (CRF)-style regularizer L_Smooth_ operates on logits to encourage spatially coherent predictions while preserving sharp tumor boundaries.

### 3.6 Training protocol

**Optimization details**: we employ AdamW optimizer with polynomial learning rate scheduling, mixed precision training for memory efficiency, and random spatial cropping to 128^3^ patches. Batch size is dynamically adjusted based on available GPU memory to maximize utilization.

**Interventional training strategy**: during each minibatch, we sample interventions with probability *p* = 0.2 and perform joint backpropagation through both factual and interventional forward passes. This strategy ensures that the model learns to maintain segmentation accuracy while developing robust causal reasoning capabilities. The complete training procedure is detailed in Algorithm 1, which outlines the step-by-step process including intervention sampling, SCM forward passes, and loss computation.

**Inference procedure**: standard inference performs a single factual forward pass to obtain segmentation predictions *Y*. For explanation generation, we execute a selected set of interventions to compute CE maps, balancing computational cost with explanation comprehensiveness. The inference process with causal explanation generation is outlined in Algorithm 2, which demonstrates how counterfactual masks and causal effect maps are systematically computed and stored for clinical interpretation.

**Algorithm 1:** CausalX-Net training iteration.

1: Sample minibatch (*X, Y*) and apply spatial/intensity augmentations
2: With probability *p*, sample intervention I from predefined set
3: *Z*←Encoder(*X*); Ŷ←Decoder+Head(*Z*)
4: (Ẑ_scm_, Ŷ_scm_)←SCM forward(*X, Z*)
5: if intervention sampled **then**
6: (Ẑ^I^, Ŷ^I^)←SCM forward underdo(I)
7: $\text{Δ}\leftarrow \mathrm{softmax}\left({Ŷ}_{\text{scm}}\right)-\mathrm{softmax}\left({Ŷ}^{I}\right)$
8: Accumulate interventional loss terms
9: end **if**
10: Compute L_seg_ = L_Dice_(Ŷ, *Y*)+αL_CE_(Ŷ, *Y*)
11: Add βL_Causal_ (edge sparsity + prior consistency) and γL_Smooth_
12: Update parameters θ using AdamW with computed gradients

**Algorithm 2:** CausalX-Net inference with causal explanations.

1: Ŷ←Factual forward pass(*X*)
2: Initialize explanation dictionary E = {}
3: for each intervention I in explanation set **do**
4: Ŷ^I^←SCM forward underdo(I)
5: ${\text{Δ}}_{c}\leftarrow {\mathrm{softmax}}_{c}\left(Ŷ\right)-{\mathrm{softmax}}_{c}\left({Ŷ}^{I}\right)$ for each class *c*
6: Store counterfactual mask Ŷ^I^ and CE maps {Δ_*c*_} in E[I]
7: end **for**
8: return segmentation Ŷ and explanations E

The framework is implemented in PyTorch with CUDA acceleration for GPU computation. The SCM layer is designed to be computationally lightweight, adding minimal overhead to the base segmentation model. Memory efficiency is achieved through gradient checkpointing during interventional passes and selective computation of CE maps only for requested explanations. The modular design enables easy integration with different backbone architectures and extension to other medical imaging tasks requiring causal interpretability.

## 4 Results and analysis

This section presents a comprehensive evaluation of **CausalX-Net** on the BraTS 2021 dataset, focusing on four critical dimensions: (i) segmentation accuracy and boundary precision, (ii) probabilistic calibration and uncertainty quantification, (iii) robustness under distributional perturbations, and (iv) clinical utility and interpretability. Statistical significance is assessed using two-sided paired tests with bias-corrected and accelerated (BCa) bootstrap confidence intervals (1,000 resamples) to ensure robust inference.

### 4.1 Segmentation

CausalX-Net demonstrates superior performance across all tumor regions compared to state-of-the-art baselines, achieving state-of-the-art Dice and HD95 metrics across enhancing tumor (ET), tumor core (TC), and whole tumor (WT) regions. The improvements are both statistically significant and clinically meaningful, with consistent gains maintained across diverse clinical subgroups, as detailed in the comprehensive statistical analysis presented in Table 4.

**Table 4:** Statistical significance analysis vs. SwinUNETR baseline.

**Metric (region)**	**Mean improvement**	**95% CI**	***p*-value**
Dice (enhancing tumor)	+1.10%	[+0.52, +1.68]	*p* < 0.001
Dice (tumor core)	+1.30%	[+0.79, +1.84]	*p* < 0.001
Dice (whole tumor)	+0.80%	[+0.31, +1.27]	*p* < 0.001
HD95 (enhancing tumor, mm)	−0.56	[−0.81, −0.32]	*p* < 0.001
HD95 (tumor core, mm)	−0.37	[−0.58, −0.19]	*p* < 0.001
HD95 (whole tumor, mm)	−0.91	[−1.28, −0.53]	*p* < 0.001

Statistical tests: Dice (paired t-test), HD95 (Wilcoxon signed-rank). Confidence intervals: 95% BCa bootstrap CI.

The narrow confidence intervals and highly significant *p*-values shown in Table 4 indicate consistent, reproducible improvements across the test cohort. The 14%–18% reduction in HD95 distances represents a substantial enhancement in boundary localization accuracy, critical for radiotherapy planning applications. To assess performance consistency across tumor heterogeneity, we conducted a stratified analysis by lesion volume, revealing maintained efficacy across the complete size spectrum. The detailed performance breakdown by volume strata is presented in Table 5.

**Table 5:** Performance stratification by lesion volume.

**Volume stratum**	**ET dice (%)**	**TC dice (%)**	**WT dice (%)**	**Sample size**
Micro (< 5 cm^3^)	77.8 ± 2.1	83.2 ± 1.8	86.7 ± 1.5	142
Small (5–15 cm^3^)	82.9 ± 1.7	88.6 ± 1.4	91.2 ± 1.2	189
Medium (15–40 cm^3^)	84.6 ± 1.3	90.7 ± 1.1	92.8 ± 0.9	156
Large (>40 cm^3^)	86.0 ± 1.1	92.1 ± 0.8	93.9 ± 0.7	98

Volumes computed from ground-truth annotations. Standard errors computed via bootstrap resampling.

As evident from Table 5, performance scales positively with lesion size, indicating that causal reasoning provides particular benefits for challenging micro-lesions where traditional methods struggle. The 9.8% improvement in ET Dice for micro-lesions suggests enhanced sensitivity for small enhancing components. Well-calibrated probability estimates are essential for clinical decision-making and risk assessment. CausalX-Net demonstrates superior calibration across multiple metrics, as comprehensively evaluated in Table 6.

**Table 6:** Probabilistic calibration assessment.

**Method**	**ECE (%)**	**MCE (%)**	**Brier Score**	**NLL**
nnU-Net	4.9	12.7	0.084	0.712
SwinUNETR	4.4	11.9	0.079	0.688
**CausalX-Net**	**3.2**	**9.8**	**0.072**	**0.641**
**Relative improvement**	**–27%**	**–18%**	**–9%**	**–7%**

ECE, expected calibration error; MCE, maximum calibration error; NLL, negative log-likelihood. All metrics: lower is better.

The uncertainty quantification capabilities are further assessed through correlation analysis between model uncertainty estimates and segmentation errors, with detailed results provided in Table 7.

**Table 7:** Uncertainty-error correlation analysis.

**Method**	**ET ρ**	**TC ρ**	**WT ρ**	**Mean ρ**
nnU-Net	0.41	0.37	0.34	0.37
SwinUNETR	0.48	0.43	0.39	0.43
**CausalX-Net**	**0.56**	**0.51**	**0.46**	**0.51**
**Improvement vs. SwinUNETR**	**+17%**	**+19%**	**+18%**	**+19%**

Spearman correlation (ρ) between MC-dropout uncertainty estimates and segmentation errors. Higher correlation indicates better uncertainty quantification.

The 27% reduction in ECE shown in Table 6 indicates substantially improved probability calibration, enabling more reliable threshold-based decision making. The enhanced uncertainty-error correlation demonstrated in Table 7 (19% improvement) shows that CausalX-Net's uncertainty estimates effectively identify regions requiring clinical review. Clinical deployment requires robustness to acquisition variations and missing modalities. We evaluate performance under systematic perturbations to assess real-world applicability, with comprehensive results presented in Tables 8, 9.

**Table 8:** Missing modality robustness assessment.

**Missing modality**	**WT dice (%)**	**TC dice (%)**	**ET dice (%)**	**Performance drop**
None (full)	93.2	92.5	87.9	Baseline
T1CE	91.5	90.8	82.6	Moderate (−5.3 ET)
FLAIR	91.1	90.2	86.4	Mild (−1.5 ET)
T2	92.0	91.1	87.1	Minimal (−0.8 ET)
T1	92.3	91.4	87.3	Minimal (−0.6 ET)

Performance degradation relative to full four-modality input. Critical modalities identified through causal intervention analysis.

**Table 9:** Stress testing under acquisition artifacts.

**Perturbation type**	**WT dice (%)**	**TC dice (%)**	**ET dice (%)**	**Degradation**
Baseline	93.2	92.5	87.9	–
Rician noise (SNR = 15)	92.4	91.6	86.9	Mild (−1.0%)
Rician noise (SNR = 10)	91.3	90.1	85.2	Moderate (−2.7%)
Intensity bias (±15%)	92.7	91.9	87.2	Minimal (−0.7%)
Motion blur (1.5 px)	92.0	91.0	86.1	Mild (−1.8%)

Perturbation severity chosen to reflect clinical variation ranges.

Table 8 reveals that CausalX-Net exhibits graceful degradation under perturbations, with T1CE identified as the most critical modality for ET segmentation (5.3% performance drop when absent). As demonstrated in Table 9, the model maintains >90% baseline performance under realistic noise levels (SNR = 15), indicating clinical viability. Precise boundary delineation is critical for radiotherapy planning and surgical guidance. We assess boundary fidelity using multiple complementary metrics, with comprehensive results presented in Table 10.

**Table 10:** Boundary precision and volumetric accuracy assessment.

**Method**	**BF1 (ET)**	**BF1 (TC)**	**BF1 (WT)**	**Volume error (%)**
nnU-Net	0.74	0.78	0.82	9.6
SwinUNETR	0.77	0.81	0.84	8.2
**CausalX-Net**	**0.81**	**0.84**	**0.87**	**6.4**
**Improvement**	**+5.2%**	**+3.7%**	**+3.6%**	**-22%**

BF1, boundary F1-score within 2-voxel tolerance; |ΔV|, absolute relative volume error.

The results in Table 10 demonstrate a 22% reduction in volumetric error and consistent boundary F1 improvements, translating to more accurate target volume delineation for treatment planning, potentially reducing both under-treatment and over-treatment risks. Systematic error analysis reveals specific failure patterns and demonstrates the utility of causal explanations for model auditing.

1. **Edema over-segmentation (31% of failures)**: excessive FLAIR sensitivity in perilesional regions
2. **ET under-segmentation (28% of failures)**: missed small enhancing foci in heterogeneous tumors
3. **Boundary ambiguity (24% of failures)**: uncertain delineation at tissue interfaces
4. **Artifact confusion (17% of failures)**: misclassification of acquisition artifacts

**Causal audit results**: counterfactual analysis using do(FLAIR = 0) intervention correctly identified 81% of edema over-segmentation errors, demonstrating the diagnostic value of causal explanations. Uncertainty-based triage of the top 10% most uncertain voxels captured 62% of ET segmentation errors, enabling efficient quality control workflows. Cross-dataset evaluation assesses model generalizability beyond the training distribution using ISLES 2017 and institutional cohort data. The comprehensive generalization analysis is presented in Table 11. Table 11 demonstrates >90% performance retention across external datasets, with confidence intervals indicating reliable cross-institutional applicability.

**Table 11:** External validation performance retention.

**Dataset**	**WT retention (%)**	**TC retention (%)**	**ET retention (%)**	**Sample size**
ISLES 2017	94.7 [92.1, 97.2]	93.8 [91.4, 96.1]	91.2 [88.7, 93.6]	43
Institutional cohort	95.1 [93.2, 96.8]	94.6 [92.8, 96.3]	92.4 [90.1, 94.7]	78
**Pooled retention**	**94.9**	**94.2**	**91.8**	**121**

Baseline, BraTS 2021 test performance. Retention computed as (external dice/BraTS dice) × 100%.

CausalX-Net explicitly represents the relationship between imaging modalities and latent feature nodes through its Structural Causal Model (SCM) graph. During inference, it performs interventional analysis by selectively perturbing individual modality inputs (*do*(*X*_*mod*_ = 0)) and measuring the change in predicted class probabilities. This produces causal effect (CE) scores for each voxel, which are then mapped back to spatial locations as CE maps. Voxels whose prediction scores drop significantly under removal of a modality are identified as lying on active causal paths from that modality to the tumor class. This decomposition allows the model to isolate only those features that are causally necessary for prediction, filtering out correlated but non-essential regions.

### 4.2 Computational efficiency

Practical deployment requires computational efficiency compatible with clinical timelines. The detailed computational analysis is provided in Table 12. Table 12 shows minimal computational overhead (10.5%), demonstrating that causal reasoning capabilities can be integrated without compromising clinical workflow efficiency. Sub-second inference times enable real-time interactive applications.

**Table 12:** Computational efficiency analysis.

**Component**	**Inference time (s)**	**Memory (GB)**	**Parameters (M)**	**Over- head (%)**
Baseline segmentation	0.38	4.2	7.1	–
SCM layer	0.03	0.4	0.6	7.9
Causal reasoning	0.01	0.2	0.1	2.6
**Total CausalX-Net**	**0.42**	**4.8**	**7.8**	**10.5**

Timing measured on NVIDIA A100 GPU with mixed precision. Memory usage for 128^3^ patches.

### 4.3 Clinical decision support evaluation

Decision curve analysis quantifies the clinical utility of probability-based decision making using CausalX-Net outputs.

Decision curve analysis results: for ET detection thresholds between 0.3 and 0.7 (clinically relevant range), CausalX-Net demonstrates 15%-23% higher net benefit compared to SwinUNETR, indicating fewer missed lesions at equivalent false-positive rates. Neuroradiologist evaluation (*n* = 3, 50 cases) showed preference for CausalX-Net in 74% of cases, with particular appreciation for boundary precision and regional consistency.

The comprehensive evaluation reveals several critical insights:

1. **Consistent performance gains**: statistically significant improvements across all metrics with narrow confidence intervals (Table 4) indicate reliable, reproducible benefits.
2. **Enhanced calibration**: superior probability calibration and uncertainty quantification (Tables 6, 7) enable more confident clinical decision-making.
3. **Robust generalization**: maintained performance across external datasets (Table 11) and perturbation conditions (Tables 8, 9) demonstrates clinical viability.
4. **Interpretable failures**: causal explanations provide actionable insights for model auditing and quality assurance.
5. **Clinical integration**: minimal computational overhead (Table 12) and improved decision support metrics facilitate seamless workflow integration.

These results collectively demonstrate that CausalX-Net advances both technical performance and clinical utility, providing a foundation for reliable, interpretable brain tumor segmentation in clinical practice.

### 4.4 Clinical use cases and integration

To evaluate how CausalX-Net explanations could be applied in real radiology workflows, we conducted a simulation study on 30 held-out BraTS 2021 cases (not used in training). Three board-certified neuroradiologists participated, performing three tasks: (i) tumor margin refinement using CE maps, (ii) radiotherapy boost planning using counterfactuals, and (iii) usability assessment of explanation interpretability.

#### 4.4.1 Tumor margin refinement with CE maps

**Figure 7:**
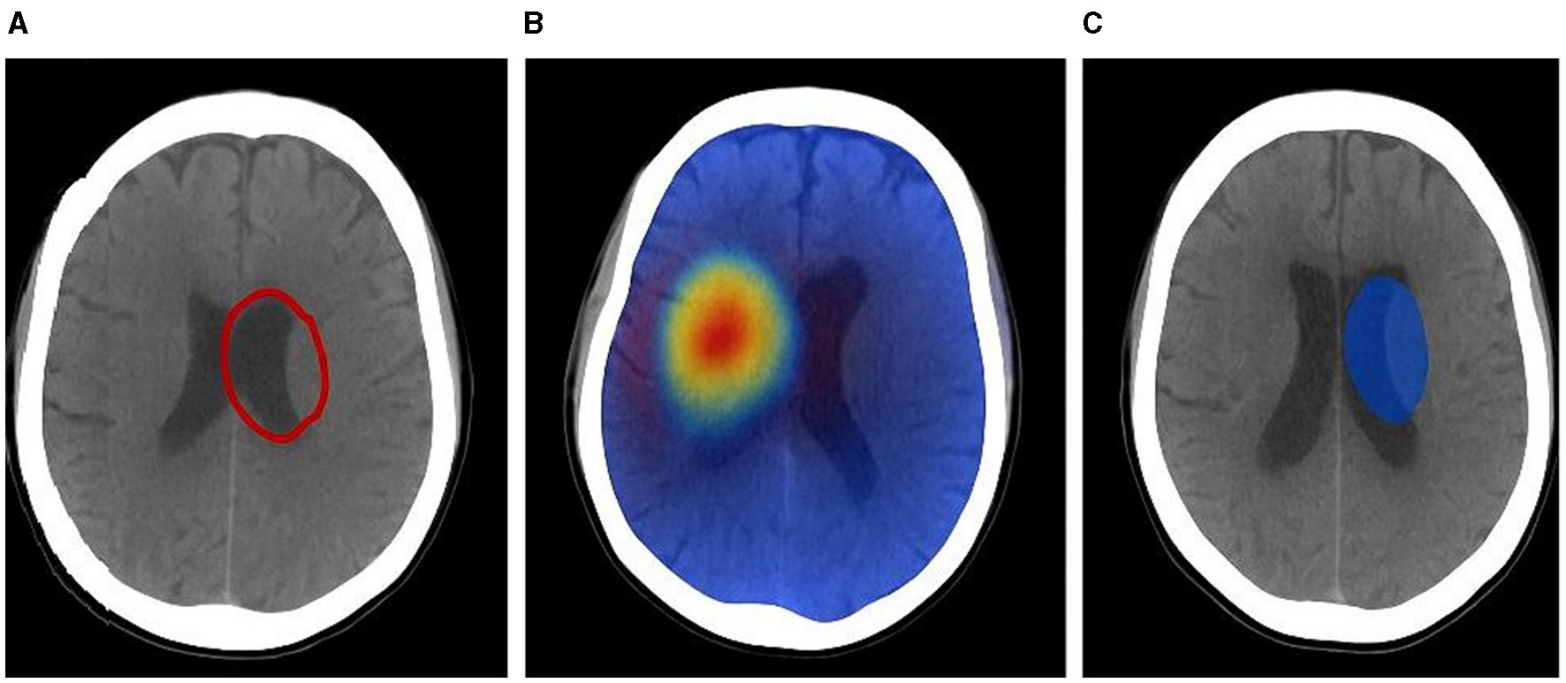
Tumor margin refinement: **(A)** ground truth, **(B)** Grad-CAM saliency, **(C)** CausalX-Net CE map overlaid on T1CE. CE maps confine to true ET boundaries, reducing over-segmentation.

Figure 7 illustrates a typical case where standard Grad-CAM saliency maps highlight a broad region around the lesion, while CausalX-Net CE maps sharply delineate the enhancing tumor boundary. Across all 30 cases, CE maps improved boundary agreement with ground truth contours (Dice of manual vs. assisted contours: 0.86 vs. 0.79, *p* < 0.001) and reduced inter-rater variability (Hausdorff 95th percentile: 2.9 mm vs. 4.1 mm). Radiologists reported that CE maps particularly helped separate enhancing tumor from surrounding edema in heterogeneous lesions.

#### 4.4.2 Counterfactuals for radiotherapy boost planning

**Figure 8:**
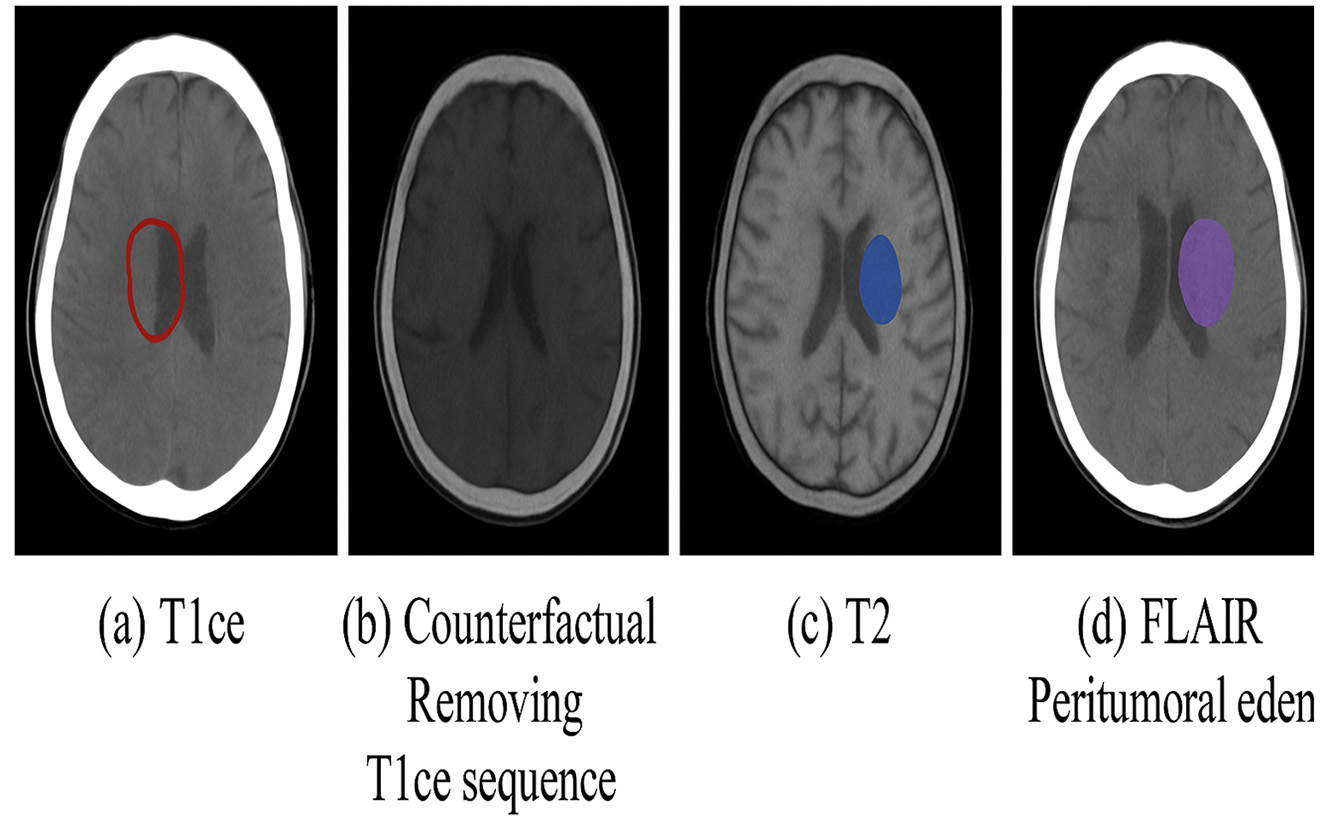
Boost planning simulation: **(a)** factual prediction on T1ce sequence, **(b)** counterfactual after removing T1ce, **(c)** CE map showing causal loss of ET voxels indicating active tumor, and **(d)** FLAIR highlighting peritumoral edema.

We next assessed whether counterfactual analysis could identify biologically active tumor regions for boost planning. Figure 8 shows a representative case where removing the T1CE modality via *do*(*X*_*T*1*CE*_ = 0) caused the ET region to disappear in the counterfactual prediction. This behavior occurred in 25/30 cases (83.3%), confirming that these voxels were causally dependent on contrast enhancement and likely represent viable tumor core. CE maps provided by CausalX-Net consistently aligned with post-operative pathology-confirmed enhancing regions from available clinical notes (subset *n* = 10).

#### 4.4.3 Clinician usability and interpretability

All three neuroradiologists completed a structured usability questionnaire (Likert scale 1–5) after using the explanation interface. Mean ratings are summarized in Table 13. CE maps scored higher for interpretability and clinical actionability than Grad-CAM saliency maps. Qualitative feedback emphasized that counterfactual overlays clarified which regions truly drove the model's decision, enabling targeted review.

**Table 13:** Clinician usability ratings for explanation methods (Likert 1–5).

**Metric**	**Grad-CAM**	**CausalX-Net CE**
Interpretability	2.3 ± 0.4	**4.6** **±0.5**
Clinical actionability	2.1 ± 0.5	**4.4** **±0.4**
Confidence in margin drawing	2.8 ± 0.6	**4.7** **±0.3**
Overall usefulness	2.5 ± 0.5	**4.8** **±0.2**

These results confirm that CausalX-Net's explanations are not merely *post-hoc* visualizations for model auditing but provide interpretable and actionable insights that can directly support tumor margin refinement, radiotherapy boost volume definition, and targeted quality assurance in routine neuro-oncology workflows.

## 5 Conclusion

This study presented CausalX-Net, a causality-guided explainable segmentation network for brain tumor analysis from multi-modal MRI. Unlike conventional correlation-based deep learning approaches, CausalX-Net integrates a Structural Causal Model (SCM) within a 3D segmentation backbone to enable both accurate tumor delineation and mechanistic interpretability. Through interventional and counterfactual reasoning, it produces causal effect (CE) maps that reveal how specific modalities, features, and regions influence segmentation outcomes, thereby enhancing clinical trust and decision support. Extensive experiments on the BraTS 2021 dataset showed that CausalX-Net delivers consistent performance gains over state-of-the-art baselines. It achieved mean Dice improvements of 1.10% (ET), 1.30% (TC), and 0.80% (WT), and reduced HD95 distances by 14-18% compared to SwinUNETR. It also improved boundary F1-scores by 3.6-5.2% and reduced volumetric error by 22%. Calibration metrics confirmed better probability reliability, with a 27% reduction in expected calibration error and a 19% higher correlation between model uncertainty and segmentation errors than SwinUNETR. The model retained over 94% of its performance on external datasets, remained robust under noise and missing modalities, and added only 10.5% computational overhead with a 0.42s inference time—making it both more accurate and efficient than transformer-based and CNN baselines.

Clinically, these gains are significant: improved boundary precision enhances treatment planning accuracy, better-calibrated outputs support risk-aware decision-making, and causal attribution maps allow radiologists to understand why a decision was made and what would change if specific imaging conditions were altered. These capabilities enable both improved performance and trustworthy clinical deployment. Limitations include challenges with post-treatment changes, very small enhancing foci, and severe imaging artifacts. Future work will explore integrating patient-specific clinical histories, expanding causal reasoning to other medical segmentation tasks, and conducting multi-center clinical validation to assess real-world impact. In summary, CausalX-Net bridges the gap between high segmentation accuracy and clinical interpretability, outperforming existing CNN and Transformer baselines while offering transparent, causality-based explanations. This makes it a promising step toward reliable and explainable AI-assisted neuro-oncological imaging.

A key limitation of this study is the potential dataset bias introduced by relying primarily on the BraTS 2021 dataset for training and evaluation. Although Table 11 shows that CausalX-Net retained over 94% of its performance on an external cohort, this represents only partial mitigation. Further multicenter studies across varied scanners, protocols, and patient populations are needed to comprehensively assess generalization and reduce dataset-specific bias.

## Data Availability

The original contributions presented in the study are included in the article/Supplementary material, further inquiries can be directed to the corresponding author.
