# Supplementary figures and images for: CausalX-Net: a causality-guided explainable segmentation network for brain tumors
Source: Front Med (Lausanne). 2025 Oct 24;12:1693603. doi: 10.3389/fmed.2025.1693603 (PMC12593452; doi:10.3389/fmed.2025.1693603)

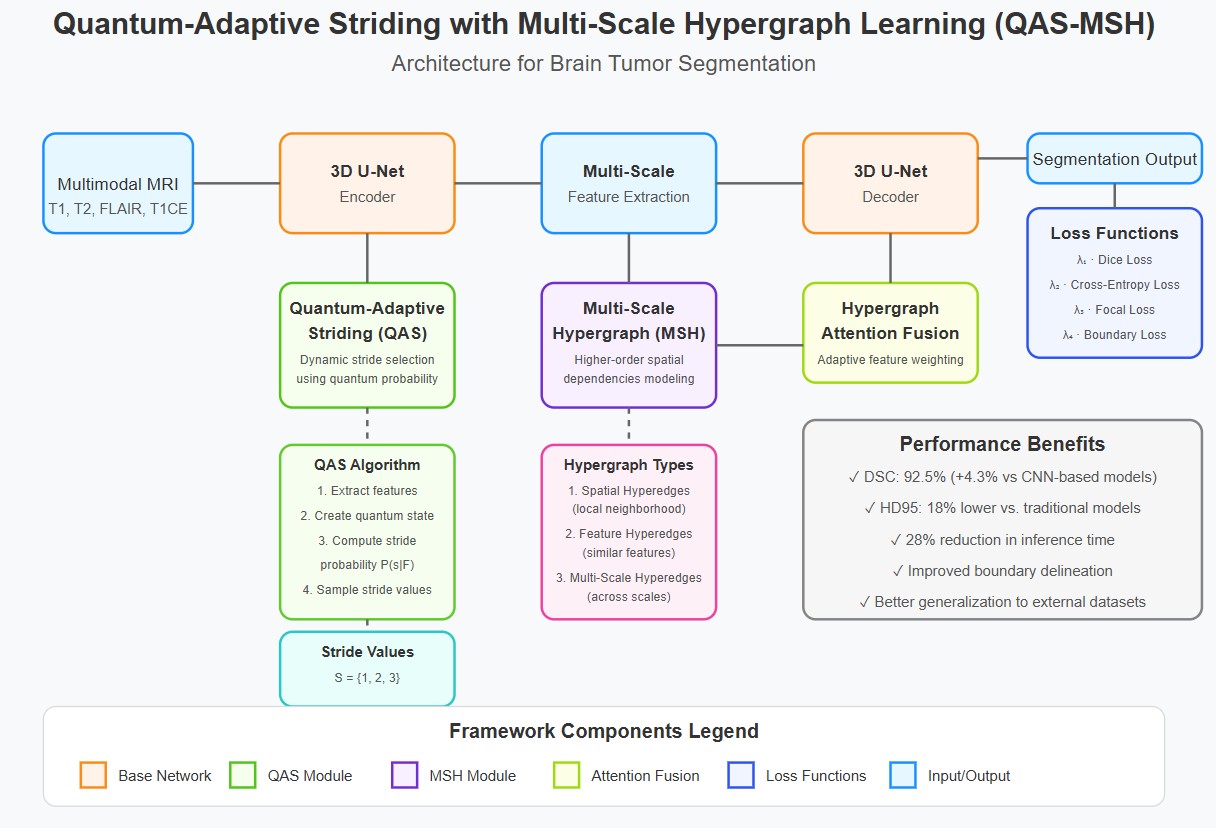

Supplement: Supplementary file 1 [file Data_Sheet_1.zip › archi.jpg]

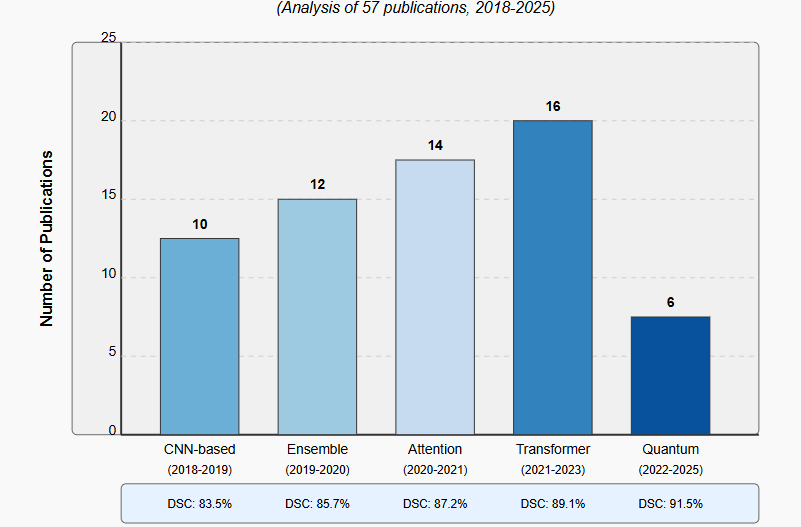

Supplement: Supplementary file 1 [file Data_Sheet_1.zip › l1.png]

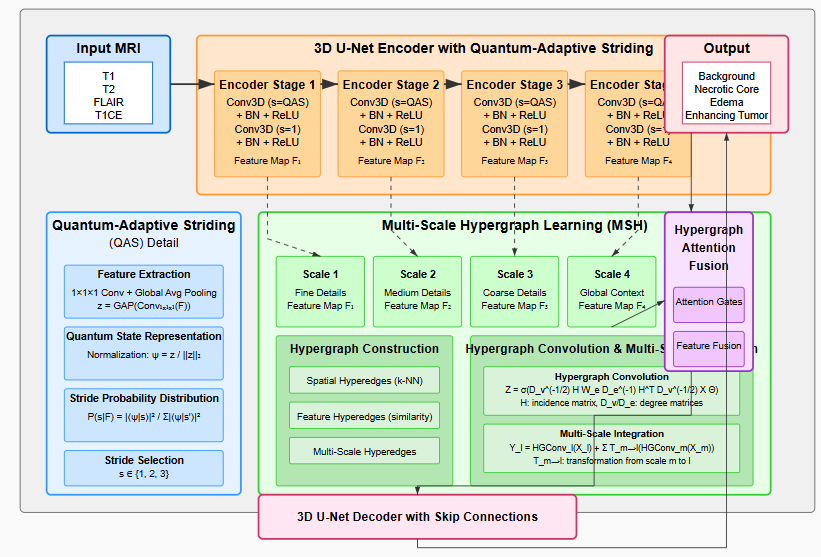

Supplement: Supplementary file 1 [file Data_Sheet_1.zip › m1.png]

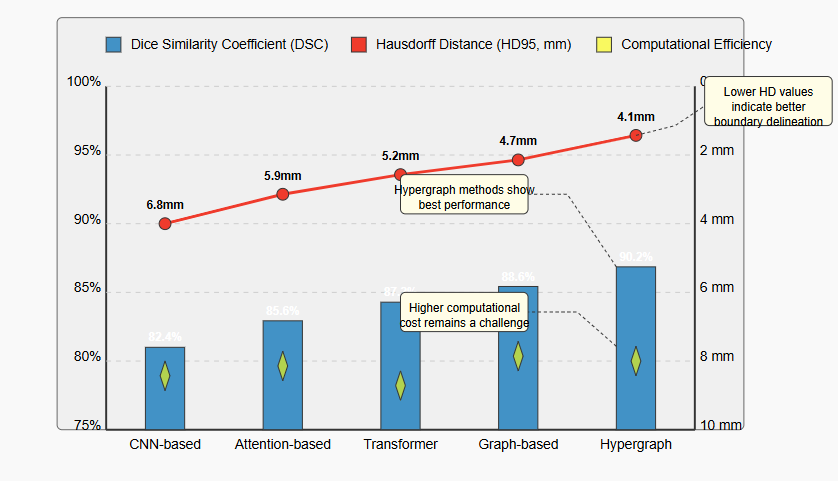

Supplement: Supplementary file 1 [file Data_Sheet_1.zip › l2.png]

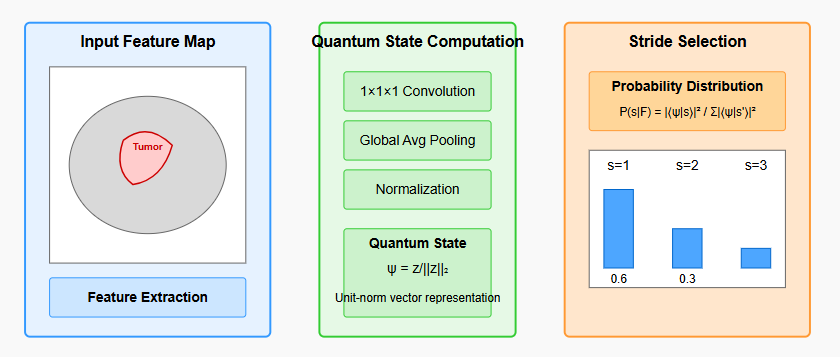

Supplement: Supplementary file 1 [file Data_Sheet_1.zip › m2.png]

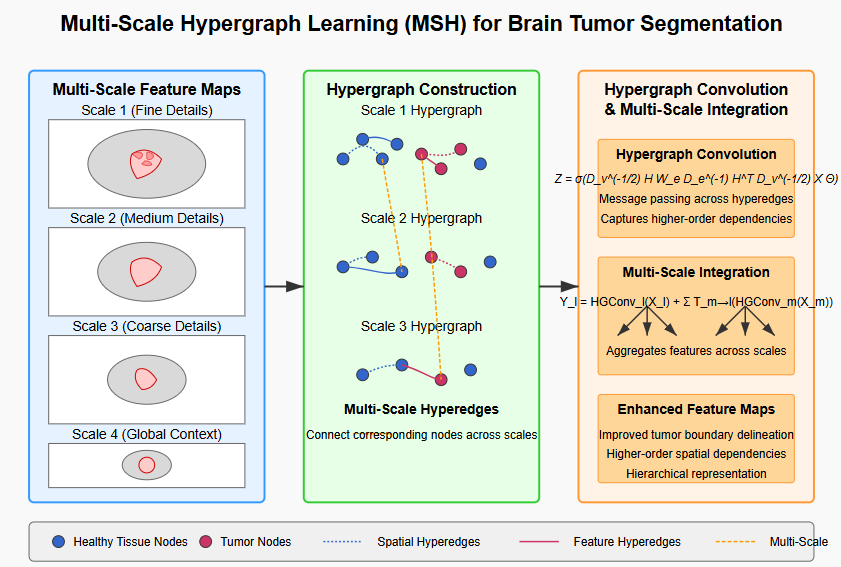

Supplement: Supplementary file 1 [file Data_Sheet_1.zip › m3.png]

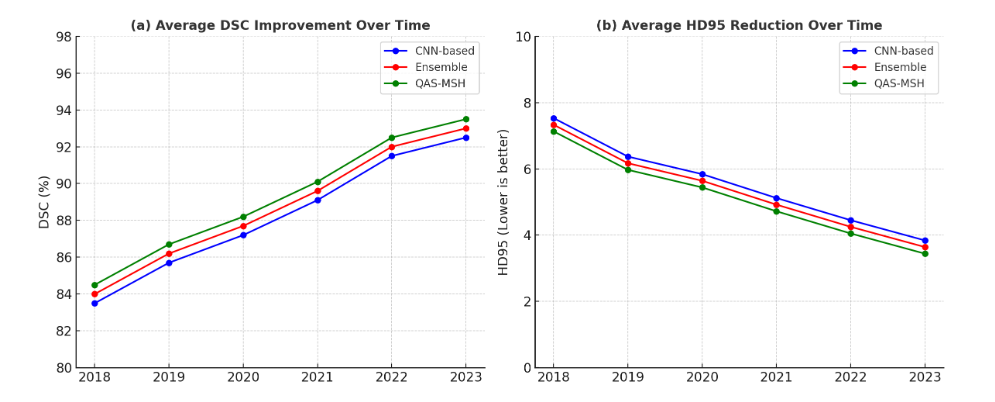

Supplement: Supplementary file 1 [file Data_Sheet_1.zip › r1.png]

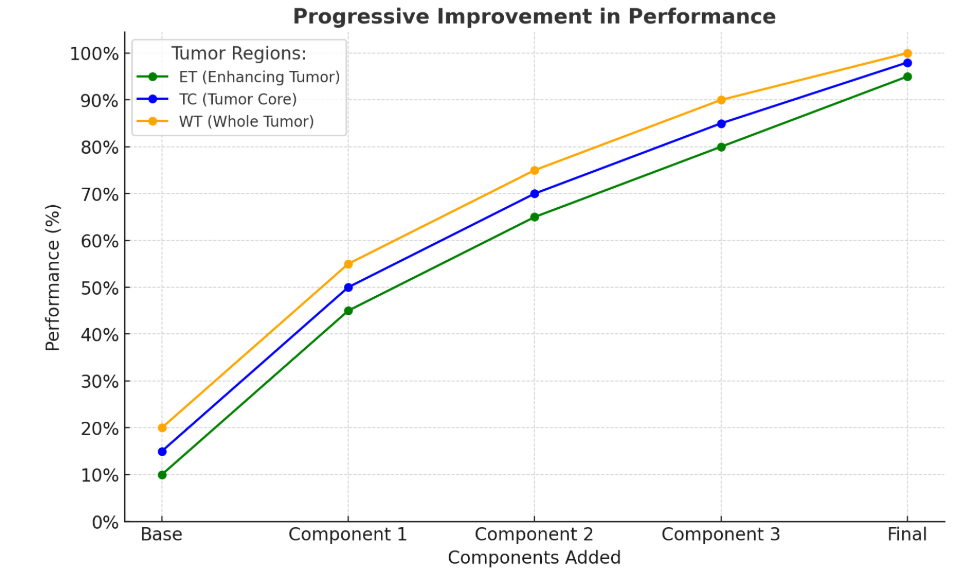

Supplement: Supplementary file 1 [file Data_Sheet_1.zip › r2.png]

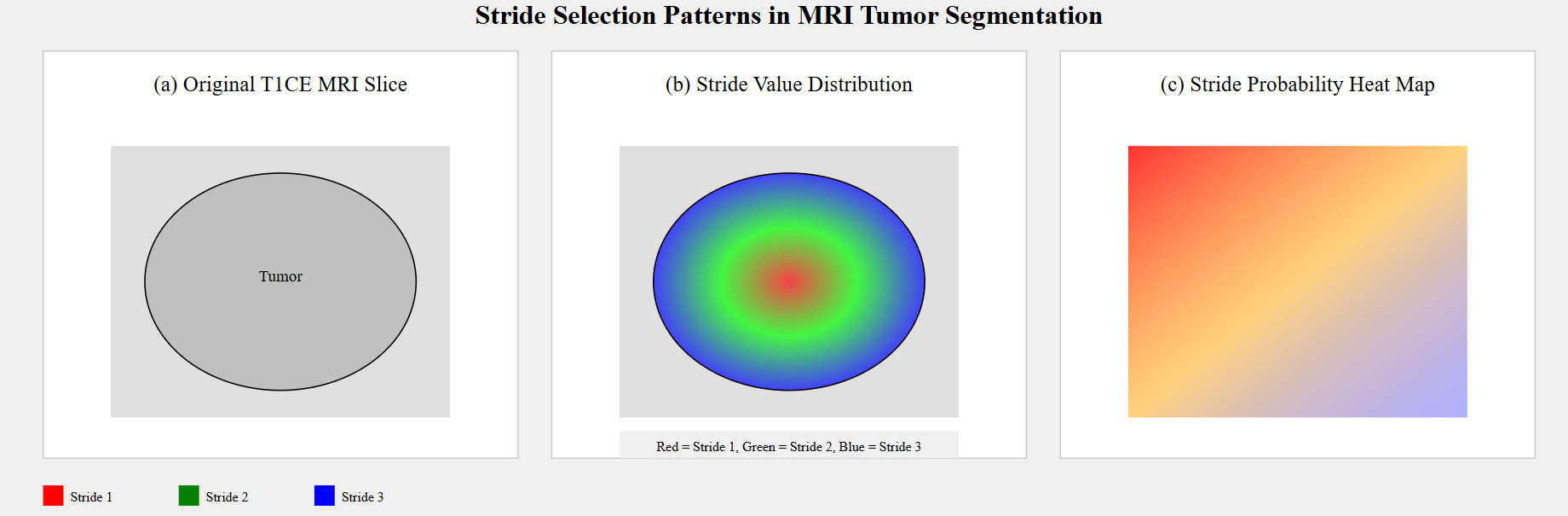

Supplement: Supplementary file 1 [file Data_Sheet_1.zip › r3.png]

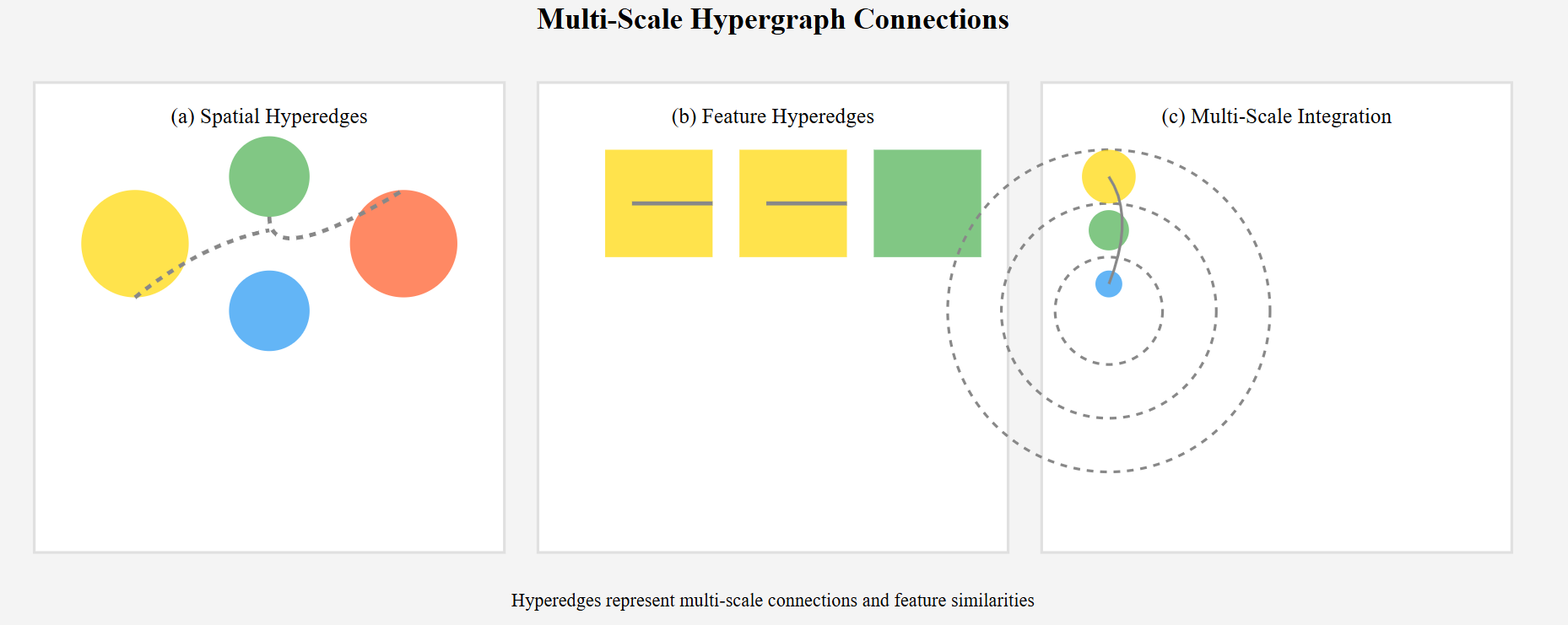

Supplement: Supplementary file 1 [file Data_Sheet_1.zip › r4.png]

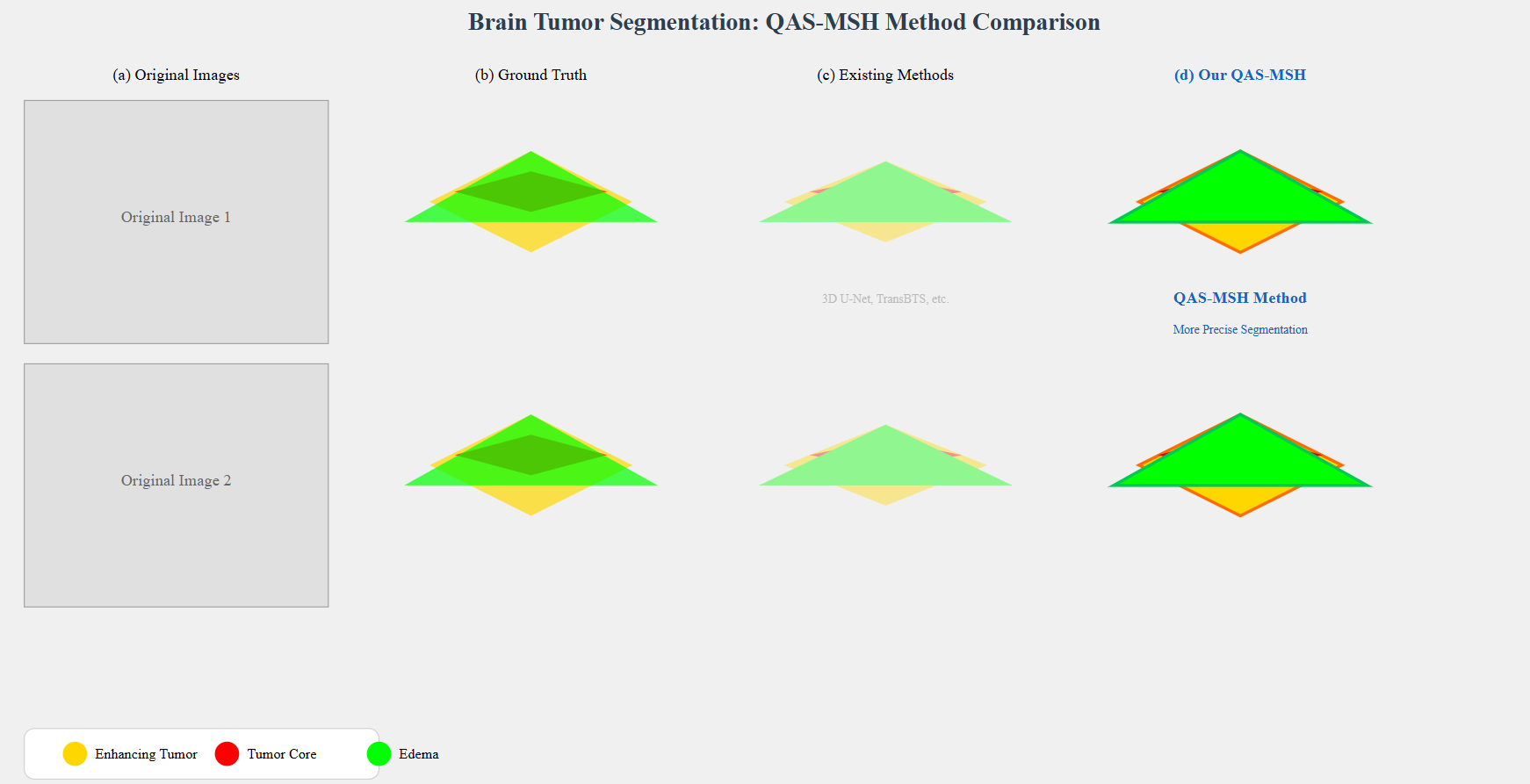

Supplement: Supplementary file 1 [file Data_Sheet_1.zip › r5.png]

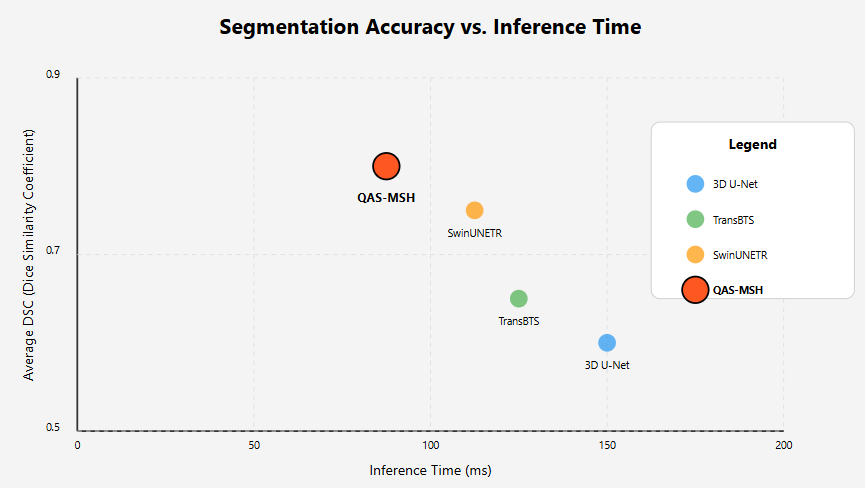

Supplement: Supplementary file 1 [file Data_Sheet_1.zip › r6.png]

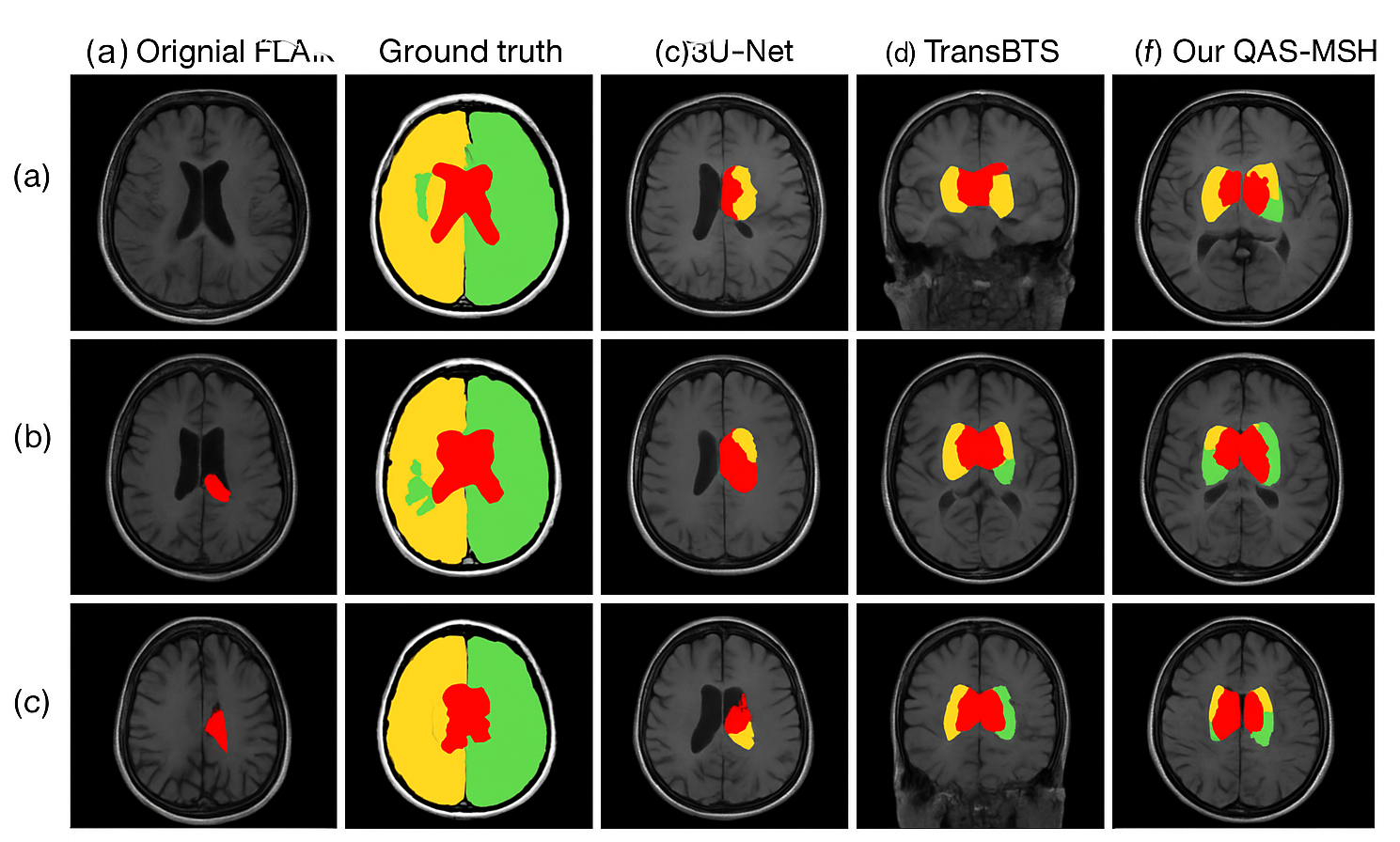

Supplement: Supplementary file 1 [file Data_Sheet_1.zip › r5v1.png]

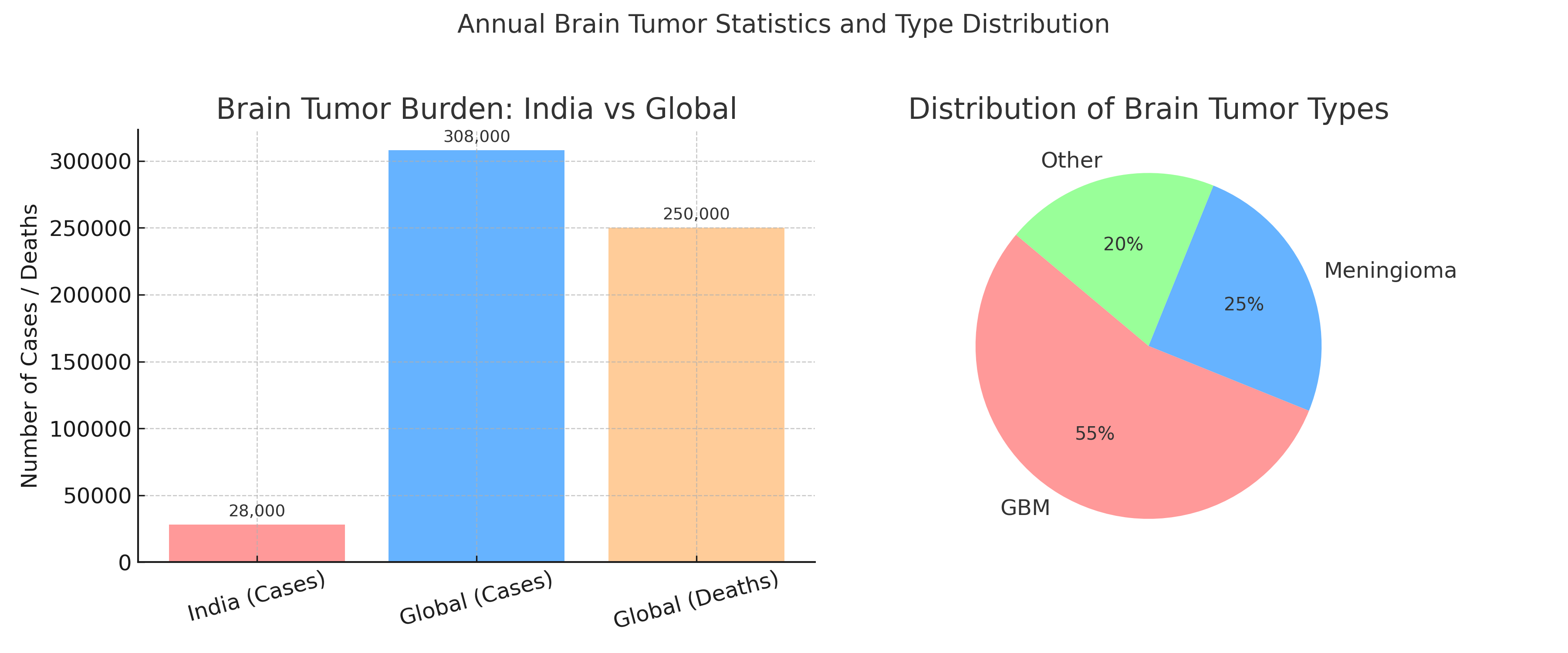

Supplement: Supplementary file 1 [file Data_Sheet_1.zip › brain_tumor_stats_fig1.png]

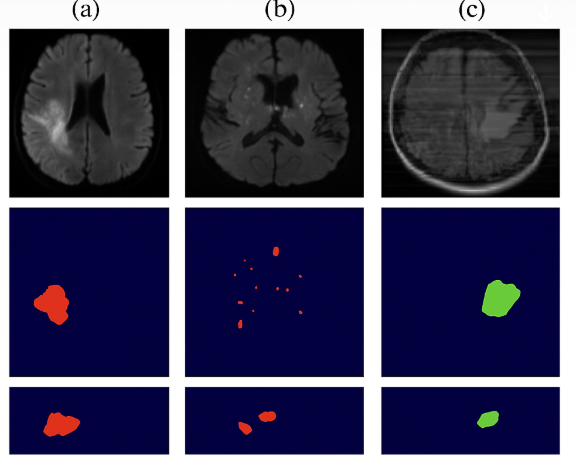

Supplement: Supplementary file 1 [file Data_Sheet_1.zip › r8.png]

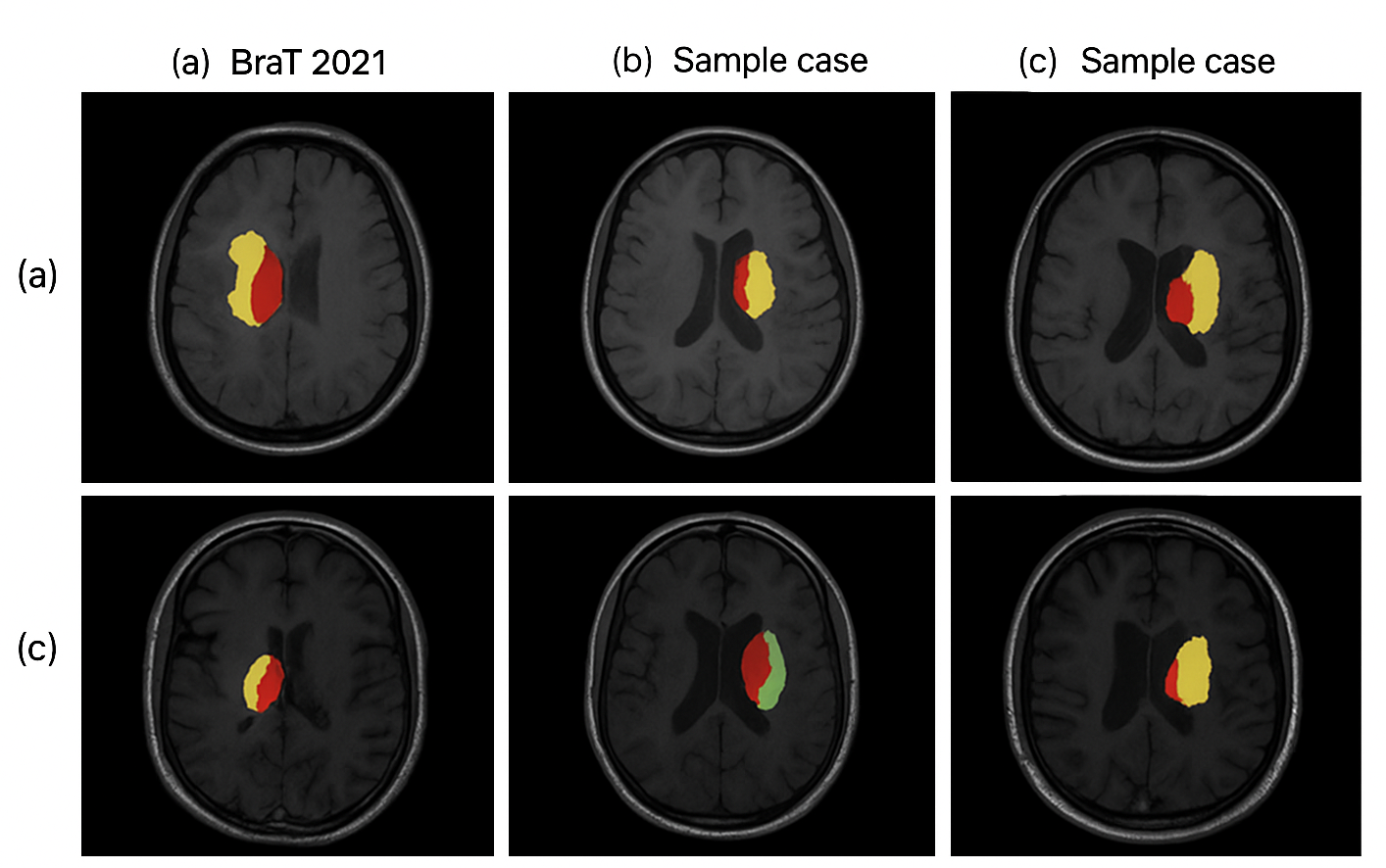

Supplement: Supplementary file 1 [file Data_Sheet_1.zip › r7.png]

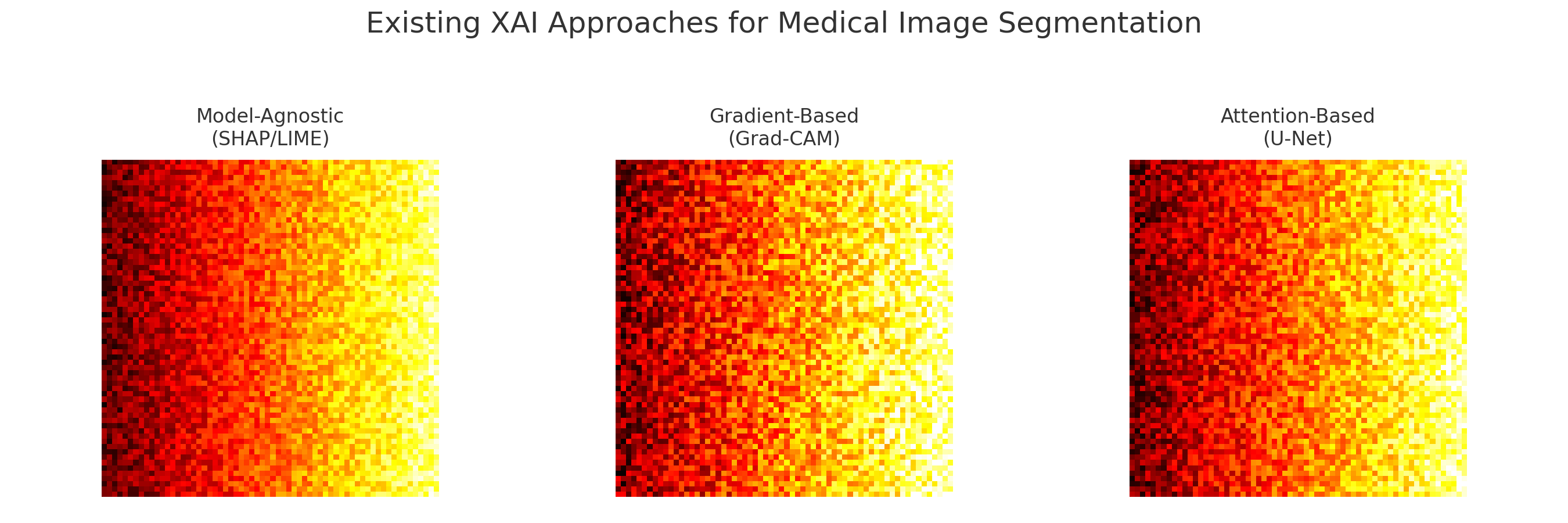

Supplement: Supplementary file 1 [file Data_Sheet_1.zip › xai_existing_methods_fig3_intro.png]

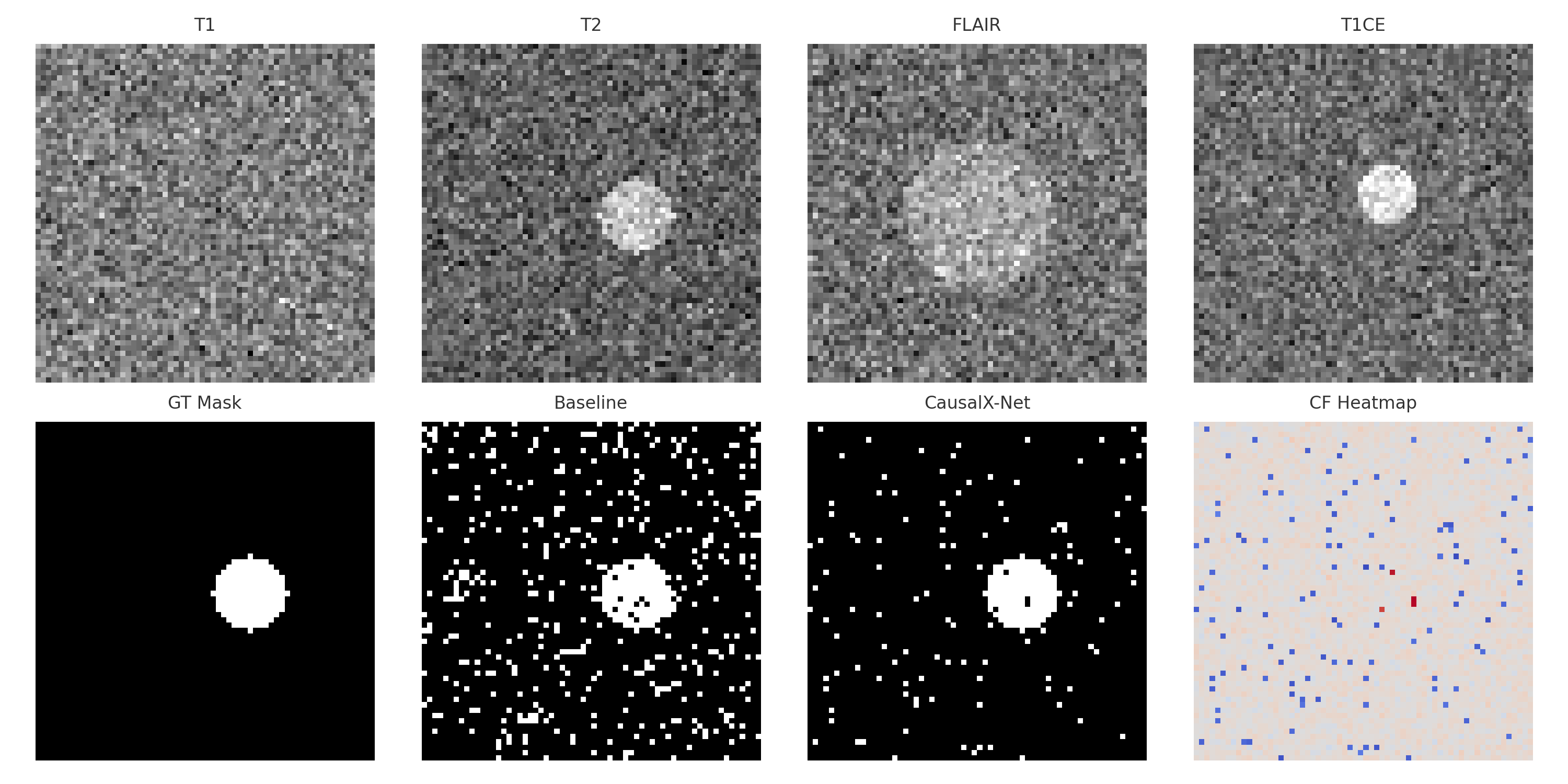

Supplement: Supplementary file 1 [file Data_Sheet_1.zip › causalxnet_results.png]

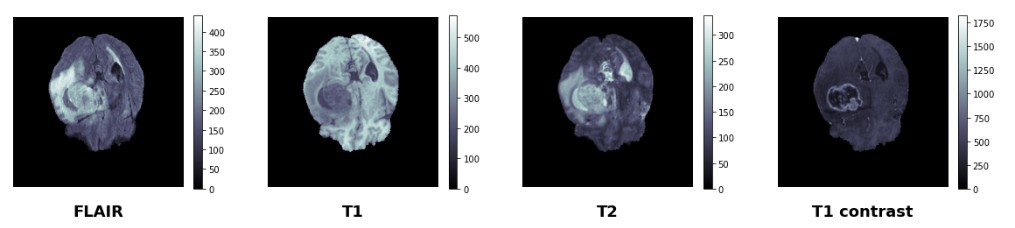

Supplement: Supplementary file 1 [file Data_Sheet_1.zip › 2.jpg]

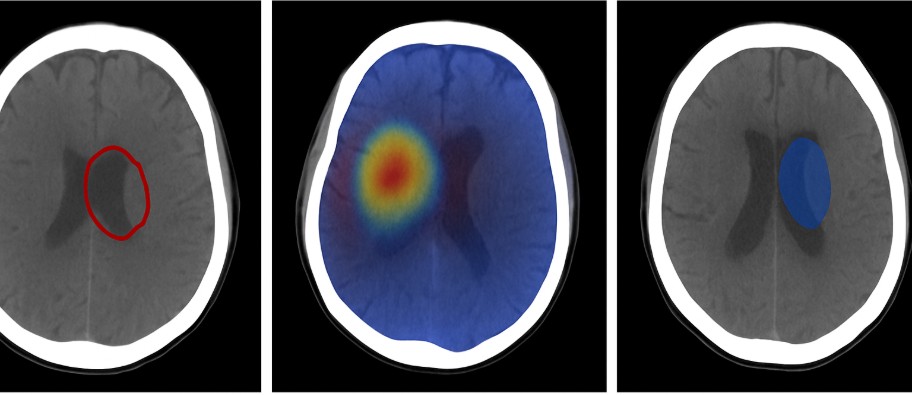

Supplement: Supplementary file 1 [file Data_Sheet_1.zip › 4.4.1.jpg]

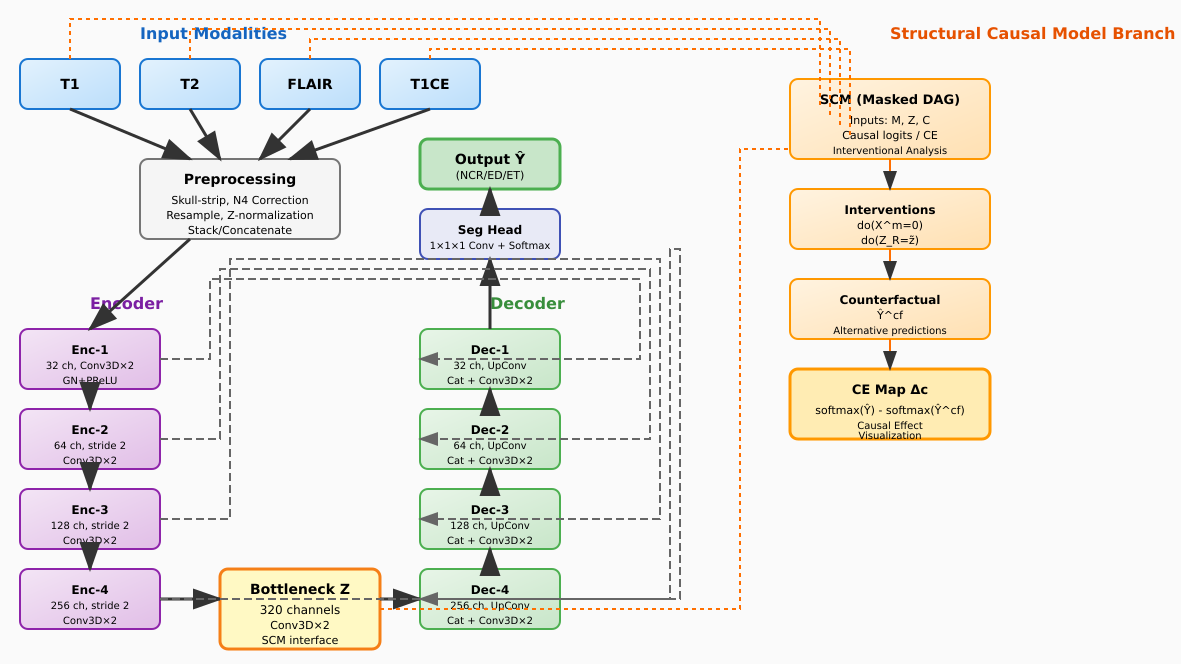

Supplement: Supplementary file 1 [file Data_Sheet_1.zip › causalx.png]

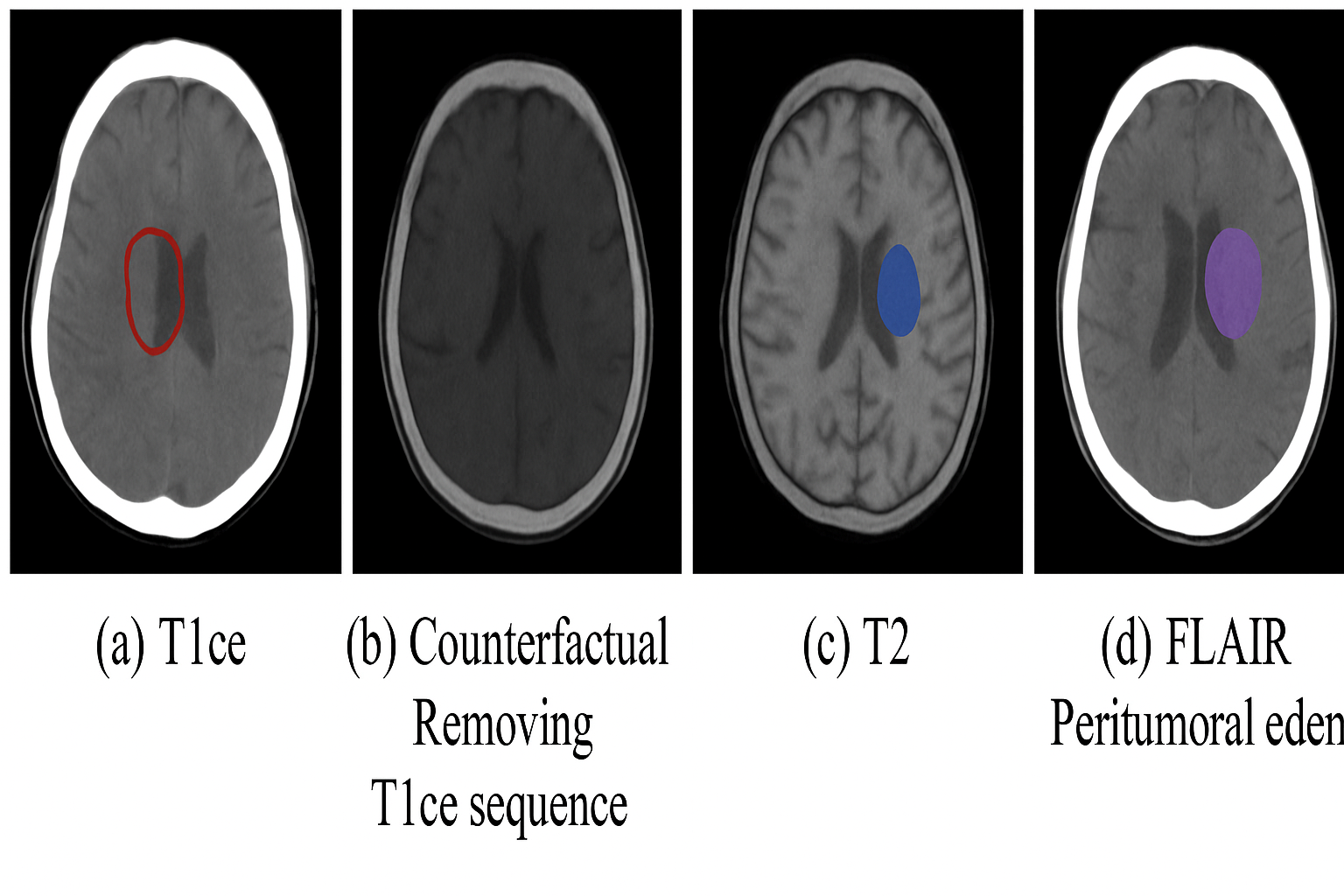

Supplement: Supplementary file 1 [file Data_Sheet_1.zip › 4.4.2.png]
